# Intermittent fasting with medium‐chain triglycerides in drug‐resistant epilepsy: A pilot crossover trial

**DOI:** 10.1002/epi.70343

**Published:** 2026-06-26

**Authors:** Wiebke Hahn, Emily Falk, Ayla Balzter, Tobias Foer, Shanin Maierhofer, Sophia Jaumann, Sascha Strehlau, Leona Möller, Panagiota‐Eleni Tsalouchidou, Katja Menzler, Lars Timmermann, Susanne Knake

**Affiliations:** ^1^ Department of Neurology, FB 20 Philipps University of Marburg Marburg Germany; ^2^ Department of Neurology Attikon University Hospital, National and Kapodistrian University of Athens Athens Greece

**Keywords:** difficult‐to‐treat epilepsy, MCT, nutrition, seizure frequency

## Abstract

**Objective:**

Ketogenic dietary therapies can reduce seizure frequency in drug‐resistant epilepsy, but adherence to the classical ketogenic diet is often poor. Intermittent fasting supplemented with medium‐chain triglycerides (MCTs) may offer a more feasible and less restrictive alternative. We assessed whether 16:8 intermittent fasting with exogenous MCT supplementation improves seizure outcomes in adults with drug‐resistant epilepsy.

**Methods:**

This prospective, single‐center, two‐period crossover pilot trial included adults (≥18 years) with drug‐resistant epilepsy and at least three seizures per month during the initial 4‐week baseline period. Participants completed two, 12‐week intervention periods—(1) 16:8 intermittent fasting plus MCT supplementation (IF–MCT) and (2) 16:8 intermittent fasting alone (IF)—separated by a 4‐week washout period. Participants were allocated 1:1 to the initial sequence using deterministic alternating allocation. The primary endpoint was seizure frequency during IF–MCT compared with IF and standard therapy during the baseline period. The trial was registered with ClinicalTrials.gov (NCT06013761).

**Results:**

Of 36 enrolled participants, 22 completed both intervention periods with evaluable seizure diary data and were included in per‐protocol analyses. During IF–MCT, two participants (9%) achieved sustained seizure freedom. In post hoc analyses, the proportion of participants achieving earlier ≥50% seizure reduction was numerically higher during IF–MCT than during IF (Breslow: *p* = .233), although not statistically significant. Concentrations of ketone bodies and octanoic and decanoic acids increased during IF–MCT, without an apparent correlation with seizure reduction.

**Significance:**

In this exploratory crossover pilot trial, intermittent fasting combined with MCT supplementation was associated with a numerical, although not statistically significant, reduction in seizure frequency compared with intermittent fasting alone in adults with drug‐resistant epilepsy. Although the study was not powered to detect definitive differences, the observed trend suggests a potential signal of efficacy. As one of the first studies to evaluate this combined dietary approach using a crossover design, the results support further investigation in larger, adequately powered multicenter efficacy trials.

**Key points**

- Adherence to ketogenic diet in adults with difficult‐to‐treat epilepsy is often poor.
- This pilot trial investigated a new nutritional strategy in drug‐resistant epilepsy: 16:8 intermittent fasting plus medium‐chain triglyceride (MCT) (IF‐MCT) supplementation.
- Seizure reduction was numerically higher during IF‐MCT than during intermittent fasting alone.
- IF–MCT demonstrated potential as a clinically relevant nutritional strategy to reduce seizure burden in drug‐resistant epilepsy.

## 1 INTRODUCTION

Epilepsy is defined by a persistent predisposition to generate epileptic seizures^1^ and is among the most common neurological disorders worldwide. Lifetime prevalence is estimated at 7.6 per 1000 individuals (95% confidence interval [CI] 6.17–9.38).^2^ Despite major advances in anti‐seizure medications (ASMs), about one‐third of patients do not achieve seizure freedom with pharmacotherapy, leading to substantial individual burden and high healthcare costs.^3^ Incidence is bimodal, with a peak in infancy and a second peak later in life, typically after age 60.^4^ With population aging, the number of adults living with drug‐resistant epilepsy (DRE) is expected to increase, underscoring the need for additional, acceptable therapeutic strategies.

Dietary therapies, particularly ketogenic dietary approaches, have re‐emerged as important options for DRE. The ketogenic diet (KD) is a high‐fat, low‐carbohydrate regimen that induces hepatic β‐oxidation and promotes the production of ketone bodies. Under conditions of low glucose availability, ketone bodies—especially β‐hydroxybutyrate (BHB) and acetoacetate—serve as alternative metabolic substrates and are also thought to exert anti‐seizure effects.^5^ The mechanisms are incompletely understood; proposed pathways include enhanced neuronal inhibition via increased γ‐aminobutyric acid signaling linked to diet‐induced alterations of the gut microbiome, and increased adenosine concentrations. Additional hypotheses include neuroprotective effects mediated by anti‐inflammatory processes, increased brain‐derived neurotrophic factor, and reduced oxidative stress.^5^

Several KD variants differ in fat content and macronutrient ratios, including the classical ketogenic diet (kKD), the medium‐chain triglyceride (MCT) diet, the modified Atkins diet, and low–glycemic index treatment. Medium‐chain fatty acids, particularly octanoic (C8) and decanoic acids (C10) used in the MCT diet, might also have direct seizure‐suppressive properties independent of ketosis.^6^ However, tolerability and long‐term adherence remain major limitations, with gastrointestinal symptoms among the most frequently reported adverse effects. Although efficacy is well established in children, evidence in adults is comparatively sparse, in part because adherence to restrictive ketogenic regimens is often poor.^7^, ^8^, ^9^, ^10^, ^11^ Equally effective dietary alternatives include the modified ketogenic diet (MKD), which is particularly popular in the United Kingdom and Ireland and recommends a daily fat intake of 60%–80%, carbohydrate intake of 5%, and unrestricted protein intake.^12^

Other nutritional approaches, such as 16:8 time‐restricted eating, have also been associated with a reduction in seizure frequency in patients with DRE.^13^

This pilot trial aimed to assess whether intermittent fasting combined with exogenous MCT supplementation reduces seizure frequency in adults with DRE, as a potentially less restrictive therapeutic alternative to established KD.

## 2 METHODS

### 2.1 Study design

This single‐center, two‐period, two‐treatment, alternating‐allocation crossover pilot trial was performed at the Epilepsy Center Hesse, University Hospital of Marburg (Marburg, Germany) between August 1, 2023, and May 31, 2025. The trial compared seizure frequency as standard care during a baseline period with seizure frequency during either intermittent fasting with medium‐chain triglyceride supplementation (IF–MCT) or during intermittent fasting alone (IF). All participants provided written informed consent before enrolment. The protocol was approved by the local ethics committee (23–92 BO) and registered at ClinicalTrials.gov (NCT06013761; study plan: https://clinicaltrials.gov/study/NCT06013761#study‐plan).

### 2.2 Participants

**FIGURE 1:**
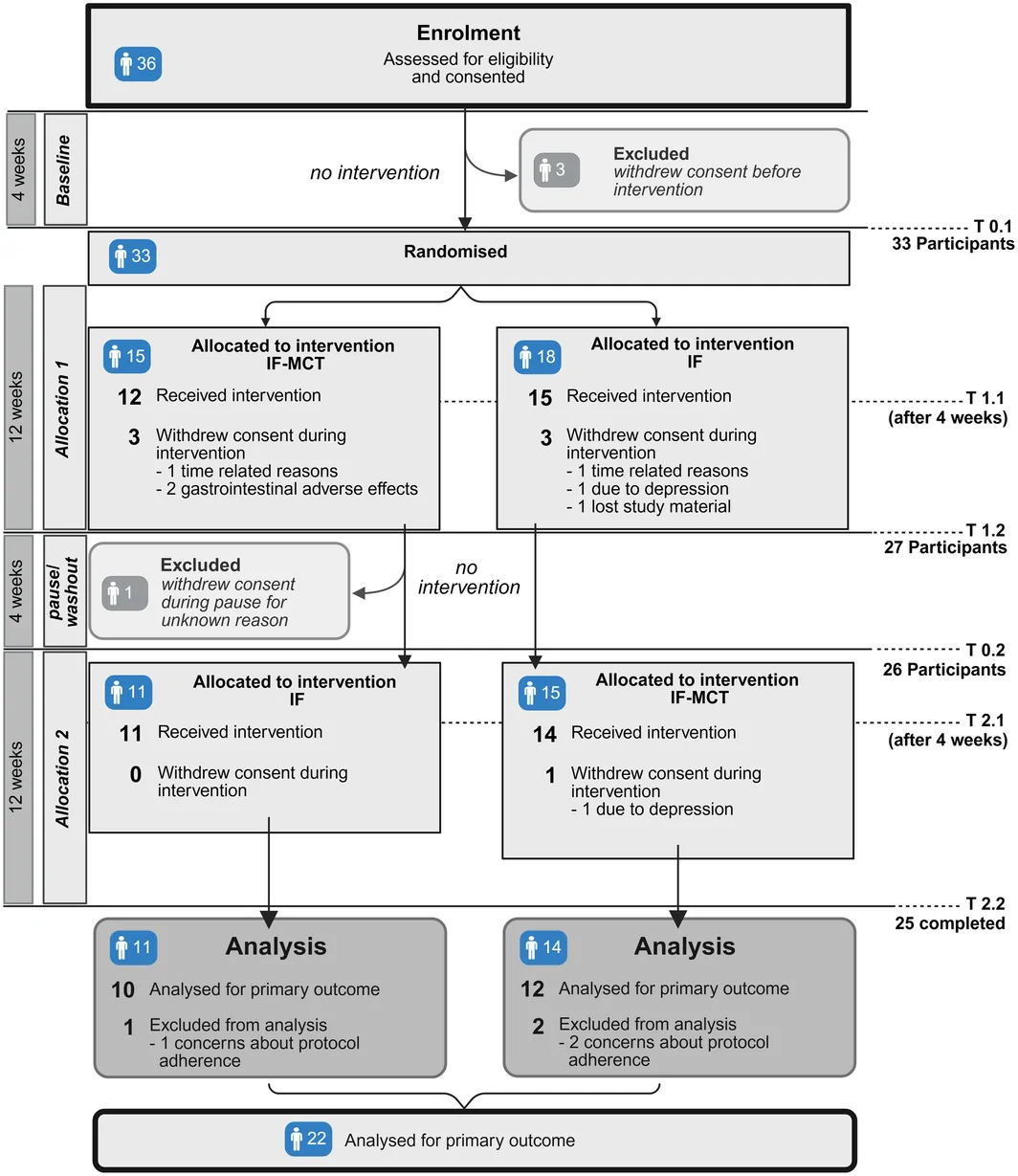
Study Design Flowchart (CONSORT) created in BioRender. Falk, E. (2026) https://BioRender.com/uwen8xk.

Thirty‐six participants were recruited from the outpatient clinic and through a regional patient organization (see Figure 1). Eligible participants were adults with DRE (defined as failure of at least two previous ASMs) and a documented seizure frequency of at least three seizures per month. This minimum baseline seizure frequency was required for study inclusion to ensure an adequate event rate for outcome assessment within the predefined observation period. Exclusion criteria were pregnancy or breastfeeding; metabolic disorders that would contraindicate a ketogenic diet (e.g., diabetes mellitus or nephrolithiasis); eating disorders; and antibiotic treatment within the preceding 3 months. ASM regimens remained unchanged throughout the study.

### 2.3 Randomization and masking

Participants were allocated (1:1) to the initial intervention sequence using a deterministic alternating scheme based on enrollment order and study identification number: participants with odd identification numbers started with IF–MCT and those with even identification numbers started with IF. Allocation was performed by the study physician, and allocation concealment was not feasible. Owing to the nature of the dietary interventions, participants and study staff were not masked to treatment assignment.

### 2.4 Procedures

After the end of a 4‐week baseline phase, participants started with a 12‐week intervention period (IF‐MCT or IF), followed by a 4‐week washout, and then crossed over to the alternative 12‐week intervention (IF or IF–MCT).

During IF, participants fasted for 16 h per day and consumed only water and unsweetened tea during the fasting window; no restrictions were imposed on food intake during the 8‐h eating window. During IF–MCT, participants followed the same fasting regimen and additionally consumed, at the end of the fasting phase, a ready‐made, commercially available formula shake with MCTs, a drink commonly used in KDs (Kanso: Keto epi/Keto epi creamy; 26 g MCT per 200 mL). The C8:C10 ratio was 50:50. The number of calories per 200 mL was 360 kcal. The fat content was 32.0 g fat (of which 26 g were MCT), with a carbohydrate content of 2 g and a protein content of 14.2 g. All patients, regardless of weight, received the same dose of MCT and shake. The MCT was titrated over 1 week, beginning with a minimal dose on Day 1 and doubling daily until a full 200 mL dose was reached from Week 2 onward.

The drinks were dispensed to participants at study visits. No dietary restrictions were applied during baseline or washout.

Participants attended seven on‐site visits: baseline, start of intervention Period 1 (T0.1), 4 weeks (T1.1) and 12 weeks (T1.2) after initiation of Period 1, after washout with start of intervention Period 2 (T0.2), and 4 weeks (T2.1) and 12 weeks (T2.2) after initiation of Period 2 (Figure 1).

Seizure frequency was recorded at each visit, neuropsychological assessments were done, and blood samples were collected for measurement of BHB, C8, and C10 concentrations in the reference laboratory. Cranial magnetic resonance imaging (MRI), electroencephalography (EEG), and cerebrovascular duplex ultrasonography were additionally performed at T0.1, T1.2, and T2.2. Weekly telephone contacts were used to support adherence. For statistical analyses, an additional 8‐week timepoint was prespecified to harmonize assessment intervals, although no on‐site visit was conducted at that time.

### 2.5 Outcomes

The primary outcome was seizure frequency during adjunct 16:8 intermittent fasting, with or without exogenous MCT supplementation, compared with standard pharmacotherapy alone (baseline), in adults with DRE. Seizures (including auras) were recorded using a standardized daily seizure diary, and adherence to fasting and eating windows was monitored with daily food logs and assessed dichotomously: adherence to the 16‐h fasting period was scored as 1, and non‐adherence as 0. Secondary outcomes (e.g., neuronal connectivity and neuropsychological outcomes) will be reported separately. Body weight at baseline and study end was recorded additionally as a variable of no primary interest (see Table S6 for details).

### 2.6 Statistical analysis

In the absence of prior studies evaluating intermittent fasting with MCT in adults, sample size determination was pragmatic, aiming to assess feasibility and generate preliminary effect estimates; a target enrollment of 28 participants was prespecified.

In this exploratory pilot study, a per‐protocol (PP) analysis was prespecified to assess the feasibility and potential efficacy of the intervention under conditions of adequate adherence, given the limited sample size and hypothesis‐generating nature of the study. Missing primary outcome data were handled by complete‐case analysis, and no imputation was performed. Complementary intention‐to‐treat analyses were conducted as sensitivity analyses and are presented in the Supporting Information.

Seizure frequency was assessed at baseline (T0.1 and T0.2), at 28 days (T1.1 and T2.1), at 56 days (8 weeks; prespecified for analysis only), and at 84 days (T1.2 and T2.2) for each intervention. A 28‐day washout period was used to minimize potential carryover effects. Within‐participant comparisons were done using the Friedman test. Between‐intervention comparisons were done using the Mann–Whitney *U* test. Spearman rank correlation was used for correlation analyses, and post hoc comparisons were done using the Wilcoxon signed‐rank test. A Bonferroni correction was applied to control for type I error due to multiple comparisons. Statistical analyses were performed using SPSS version 30 (IBM Corp., 2024).

### 2.7 Role of the funding source

This study was financed by an unrestricted donation from Kanso to the Otto Loewi Foundation (a non‐profit association supporting research, therapy, and training in neurology). The funder of the study had no role in study design, data collection, data analysis, data interpretation, or writing of the report.

## 3 RESULTS

Between August 1, 2023, and May 31, 2025, a total of 36 patients meeting the inclusion criteria were enrolled in the IF‐MCT study at the university outpatient clinic.

### 3.1 Patient characteristics and dropouts

Three participants withdrew consent before the intervention. Eight discontinued the intervention: two because of gastrointestinal adverse events, two because of time constraints, one because of loss of study materials, two because of recurrence of depressive symptoms unrelated to study procedures, and one for unspecified reasons. Of the 25 participants who completed the trial, three were excluded from the analysis owing to substantial concerns about adherence to study procedures (Figure 1). Thus, 22 participants were included in the final analysis: 19 were female and 3 were male; 16 had focal epilepsy, 2 had generalized epilepsy, and 4 had epilepsy of undetermined type (Table 1). ASM regimens remained unchanged throughout the study for all but one participant, whose treatment was modified because of seizure clustering during the first intervention period (Table 1).

**TABLE 1:** Patient characteristics.

Patient	Age	Sex	Start sequence	Diagnosis and epileptogenic zone	Current seizures types and medical relevant factors	Current anti‐seizure medication (ASM)
3	57	F	B	Focal epilepsy (right temporal due to hippocampal sclerosis)	I: focal seizure with impaired consciousness, prior selective amygdala hippocampectomy, right	BRV, LTG
4	56	F	A	Focal epilepsy (left parietal; suspected lesion left temporal)	I: focal seizure with impaired consciousness	BRV, LTG, STP
5	39	F	B	Focal epilepsy (right temporal due to hippocampal sclerosis)	I: epigastric aura II: myoclonus III: bilateral tonic–clonic	LTG
6	33	F	A	Absence epilepsy (generalized; most likely genetic)	I: absence	LTG + LEV
7	49	F	B	Focal epilepsy (left occipital; unclear etiology)	I: focal seizure with preserved consciousness, vagus nerve stimulator	LTG, LCM, CBM
8	37	F	A	Focal epilepsy (right temporal due to dysembryoplastic neuroepithelial tumor (DNET) of the right insula; WHO I)	I: myoclonus, prior partial resection of the DNET	LEV, LTG
9	66	F	B	Focal epilepsy (left frontal; unclear etiology)	I:focal seizure with preserved consciousness	LEV (switching to BRV after first study phase due to seizure accumulation), LTG
13	62	F	B	Focal epilepsy (right temporal due to prior irradiation of a cerebral AV angioma and possibly irradiation‐induced hippocampal sclerosis in the right, small arachnoid cyst in the right temporal pole)	I: focal seizure with impaired consciousness	ESL, CBM
15	46	F	B	Focal epilepsy (unclear epileptogenic zone; most likely genetic)	I: myoclonus	LCM, BRV, CBZ, STP
16	21	M	A	Absence epilepsy (generalized; most likely genetic)	I: absence II: bilateral tonic–clonic	BRV, LCM, PER
17	28	F	B	Focal epilepsy (left frontal; unclear etiology; most likely heterotopia on the left)	I: bilateral tonic–clonic II: focal seizure with preserved consciousness III: focal seizure with impaired consciousness, EASEE‐stimulation system	BRV, LCM, CBM
18	54	F	A	Focal epilepsy (left temporal due to DNET DD ganglioglioma)	I: focal seizure with preserved consciousness II: focal seizure with impaired consciousness	BRV, LCM, PER
19	43	F	B	Focal epilepsy (bitemporal; structural lesion on the left temporal side; most likely DNET)	I: focal seizure with preserved consciousness II: focal seizure with impaired consciousness	LCM, BRV
20	27	F	A	Focal epilepsy (frontal; unclear etiology)	I: bilateral tonic–clonic II: myoclonus	none, based on patient wish
21	62	F	B	Most likely temporal lobe epilepsy (unclear epileptogenic zone and etiology)	I: focal seizure with preserved consciousness II: focal seizure with impaired consciousness III: epigastric aura IV: bilateral tonic–clonic	LTG, PGB, CBM, PER
26	37	F	A	Unclear (suspicious of left temporal; unclear etiology)	I: focal seizure with impaired consciousness II: bilateral tonic–clonic	ZNS
28	31	F	A	Unclear (suspicious of right frontal; unclear etiology)	I: focal seizure with preserved consciousness II: bilateral tonic–clonic	VPA
30	24	F	A	Most likely frontal lobe epilepsy (unclear epileptogenic zone and etiology)	I: focal seizure with impaired consciousness, vagus nerve stimulator	LCM, PER + BRV
32	36	F	A	Focal epilepsy (most likely on the right frontal and occipital; suspicious of fronto‐mesial focal cortical dysplasia on the right)	I: epigastric aura II: focal seizure with impaired consciousness III: bilateral tonic–clonic	BRV, TPM
33	56	M	B	Focal epilepsy (right frontal, due to cystic defect temporoparietooccipital on the right)	I: bilateral tonic–clonic, vagus nerve stimulator	LTG, BRV, TPM
34	43	F	A	Focal epilepsy (right temporal due to cavernomatosis cerebri)	I: focal seizure with impaired consciousness II: bilateral tonic–clonic, prior resection of two temporal cavernomas	BRV, TPM, LCM
35	46	M	B	Focal epilepsy (bitemporo‐parieto‐occipital due to double cortex syndrome)	I: focal seizure with preserved consciousness II: focal seizure with impaired consciousness, vagus nerve stimulator	LTG, STM, CBM

Abbreviations: BRV, brivaracetam; CLB, clobazam; CNB, cenobamate; DNET, dysembryoplastic neuroepithelial tumor; ESL, eslicarbazepine; F, female; LCM, lacosamide; LEV, levetiracetam; LTG, lamotrigine; M, male; PER, perampanel; PGB, pregabalin; sequence A, IF first; Sequence B, IF–MCT first; STM, sultiame; STP, stiripentol; TPM, topiramate; VPA, valproate; ZNS, zonisamide.

### 3.2 Adverse effects

All participants experienced transient gastrointestinal side effects, predominantly fullness and mild nausea, during the titration phase. These symptoms were alleviated by emulsification of the shake or the consumption of gastrointestinal‐soothing teas. Notably, adverse effects were self‐limiting and diminished with continued MCT intake.

### 3.3 Per‐protocol analysis

Due to dropouts or exclusion, 22 patients were finally included in the PP analysis. Among these, two achieved sustained seizure freedom during IF–MCT, at T1.2 compared with the 28‐day baseline period during which patients received standard pharmacotherapy only. Both IF–MCT and IF were administered as add‐on interventions (Table 2).

**TABLE 2:** Sum of overall seizures during ketogenic intermittent and intermittent fasting.

Patient	Sz sum baseline 4 weeks	Sz sum IF MCT 4 weeks	Sz sum IF MCT 8 weeks	Sz sum IF MCT 12 weeks	Sz sum pause (washout)	Sz sum IF 4 weeks	Sz sum sz IF 8 weeks	Sz sum IF 12 weeks
3	3	0	1	0	1	0	0	0
4	8	11	9	10	11	8	10	11
5	8	5	0	1	10	5	6	2
6	420	345	300	340	275	285	325	320
7	12	11	12	10	12	9	11	9
8	20	15	19	17	10	17	14	12
9	5	15	16	25	25	28	15	19
13	8	5	8	5	5	4	3	4
15	8	31	36	23	25	29	19	16
16	58	20	10	10	12	41	32	16
17	7	2	2	3	1	2	1	2
18	11	8	12	11	12	9	6	8
19	8	6	4	12	7	7	5	8
20	4	2	0	2	5	3	0	2
21	7	11	7	9	6	3	8	4
26	6	3	2	6	2	9	0	10
28	94	216	196	131	157	170	255	185
30	12	9	13	20	10	13	10	7
32	51	41	52	43	44	50	42	39
33	3	3	1	0	1	0	1	2
34	3	5	5	3	3	8	5	5
35	10	12	7	6	7	14	9	7

Abbreviations: IF, intermittent fasting; IF–MCT, intermittent fasting plus medium‐chain triglyceride supplementation; pause, washout period; sz, seizures.

For seizure frequency during IF–MCT and IF at Weeks 4, 8, and 12 in comparison to baseline condition (T0.1), we found no evidence of within‐participant differences (Friedman test; *p* = .183). In the between‐group analysis comparing seizures with regard to the starting condition (starting with IF–MCT vs starting with IF) at Weeks 4, 8, and 12, we found no evidence of differences (Mann–Whitney *U* test; Table S1). The results of the intention‐to‐treat (ITT) analyses were consistent with the PP findings, with no statistically significant effects observed. Detailed ITT results, including a comprehensive overview of all participants and missing data patterns, are provided in the Supporting Information (Table S4a–c).

**FIGURE 2:**
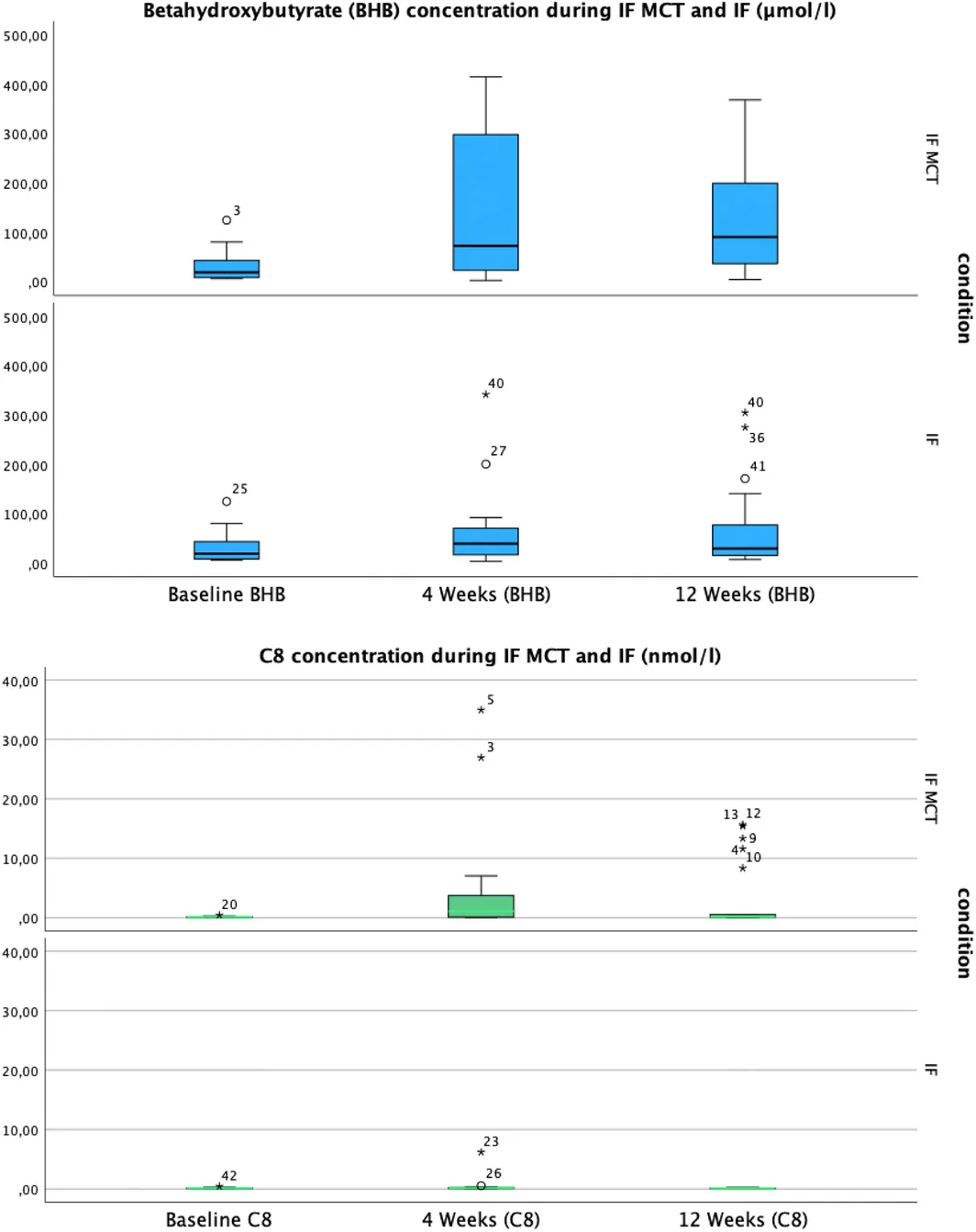
Boxplots of beta‐hydroxybutyrate (BHB), octanoic acid (C8), and decanoic acid (C10) concentration in the overall study duration.

Adherence to IF did not differ across study phases (*p* = .811). Across study conditions, concentrations of BHB differed significantly (Friedman test; *p* < .001). Post hoc analyses with Wilcoxon exact ranc test revealed that this effect was driven largely by higher concentrations after 4 weeks (*p* = .002) and 12 weeks (*p* < .001) of IF–MCT than at baseline. This effect could not be observed for IF. Concentrations of C8 also differed across conditions (Friedman test; *p* < .001), driven largely by higher concentrations after 4 weeks (*p* = .001) and 12 weeks (*p* = .011) of IF–MCT than at baseline or IF. Similarly, concentrations of C10 differed across conditions (Friedman test; *p* < .001), driven largely by higher concentrations after 4 weeks (*p* < .001) and 12 weeks (*p* = .011) of IF–MCT than at baseline or IF. We found no evidence of corresponding changes for C8 and C10 after 4 or 12 weeks of IF alone (Figure 2). Seizure frequency at 4, 8, and 12 weeks of intervention (IF–MCT or IF) was not associated with concentrations of BHB, C8, or C10 after adjustment for multiple testing (Table S2a).

**FIGURE 3:**
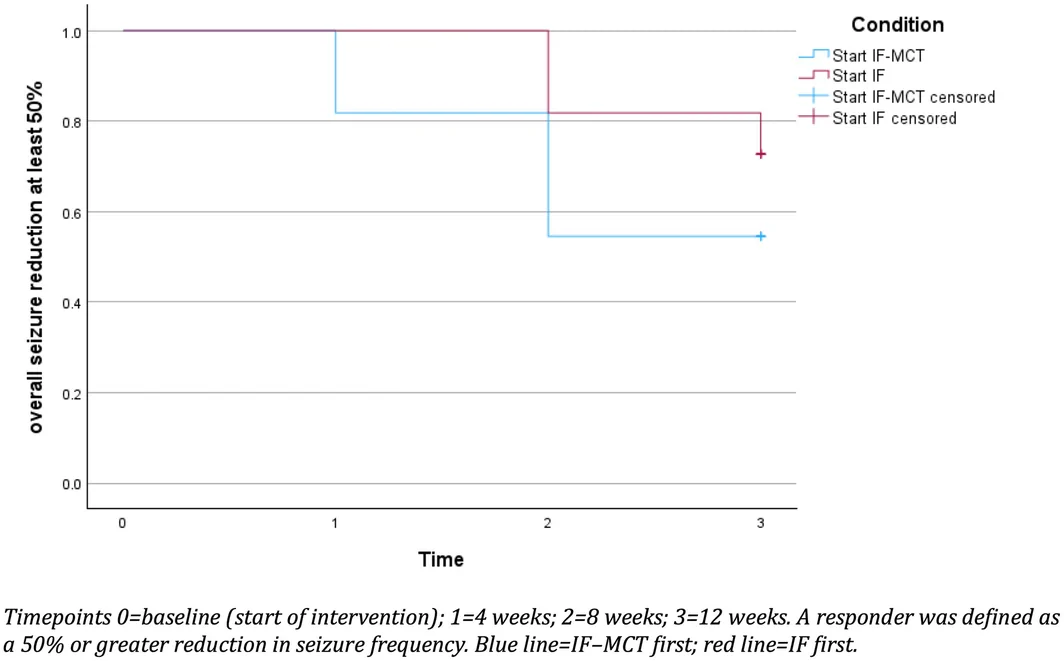
Kaplan–Meier curve regarding an at least 50% overall seizure reduction.

Post hoc analyses showed that 8 (36.6%) of 22 participants achieved a 50% or greater reduction in seizure frequency during the intervention periods. This effect was driven mainly by the IF–MCT period, during which five participants achieved a 50% or greater seizure reduction (Figure 3; for details see Table S3a,b). In addition to the clearer seizure‐suppressive effect in the IF‐MCT condition, a Kaplan–Meier curve also indicated an earlier effect during ketogenic intermittent fasting (Breslow: *p* = .233). Achieving a 50% or greater seizure reduction at 4, 8, and 12 weeks of intervention (IF–MCT or IF) was not associated with concentrations of BHB, C8, or C10 after adjustment for multiple testing (Table S2b).

## 4 DISCUSSION

In this single‐center, prospective, longitudinal crossover pilot trial, we assessed whether 16:8 intermittent fasting supplemented with MCTs (IF–MCT) reduced seizure frequency in adults with DRE. Of 36 participants who provided written informed consent, 25 completed the trial and 22 were included in the PP analyses. For the primary outcome, seizure frequency during IF–MCT did not differ significantly from intermittent fasting alone in either within‐participant or between‐sequence analyses. However, post hoc analyses suggested a faster and higher likelihood of achieving a 50% or greater reduction in seizure frequency during IF–MCT (Breslow: *p* = .233).

The trial did not reach the prespecified sample size of 28 participants, largely because of a drop‐out rate of 39% (14/36), corresponding to a PP retention of 61% (22/36). This level of retention is consistent with adherence rates reported for ketogenic dietary therapies in adults with epilepsy,^7^, ^11^, ^14^ including modified Atkins–based approaches, and likely reflects the sustained and restrictive nature of these dietary interventions as well as common preferences for carbohydrate‐rich foods.^11^, ^14^ Of note, discontinuation due to clinically relevant adverse effects, particularly gastrointestinal adverse effects, were uncommon (6%, 2/36), suggesting that the drop‐out rate was not driven predominantly by tolerability.

Although underpowered to detect statistically significant differences in seizure frequency, exploratory analyses suggested a signal favoring IF–MCT: 8 of 22 participants (36.6%) achieved a 50% or greater seizure reduction, and 2 participants achieved sustained seizure freedom during IF–MCT. Kaplan–Meier analyses suggested that this effect was driven primarily by the IF–MCT condition. These findings are broadly consistent with evidence in adults showing that adjunctive ketogenic dietary therapy (typically the modified Atkins diet) can achieve a 50% seizure reduction,^7^, ^14^ while potentially avoiding the need for sustained dietary restriction across the entire day. IF–MCT might therefore represent a less restrictive alternative to conventional KDs for some adults with DRE.

Seizure frequency was not associated with blood concentrations of BHB, octanoic acid (C8), or decanoic acid (C10) (Table S2). Although IF–MCT increased BHB concentrations compared with IF (peak value IF‐MCT: 0.13 mmol vs peak value IF: 0.06 mmol; Figure 2), the degree of ketosis might have been insufficient. A ketogenic metabolic state, especially when induced by the modified Atkins diet, is associated with ketone body concentrations of 0.5–3.0 mmol/L, in contrast to intermittent fasting alone, which generally yields ketone levels below 0.5 mmol/L after 14–24 h of fasting.^15^ However, available evidence does not support a consistent association between BHB concentrations and seizure reduction in DRE.^6^, ^16^, ^17^, ^18^

In animal models, anti‐seizure effects of MCTs have been attributed to direct and selective inhibition of α‐amino‐3‐hydroxy‐5‐methyl‐4‐isoxazolepropionic acid (AMPA) receptors, which have a central role in seizure propagation.^6^, ^19^ In vitro studies have also linked MCTs to increased mitochondrial citrate synthase activity, as well as increased complex I and catalase activity.^6^, ^20^ Previous studies reporting a negative correlation between MCT concentrations and seizure frequency used substantially higher doses (80 g/day), but with high discontinuation rates due to gastrointestinal adverse effects.^21^ Overall, the mechanisms underlying the anti‐seizure effects of ketogenic dietary therapy—and particularly those of MCTs—remain incompletely understood, and might involve metabolic pathways more complex than previously assumed.^6^, ^20^

This study has limitations. Methodological limitations include the exploratory pilot design and insufficient power to detect statistically significant differences in seizure frequency, largely because the prespecified sample size could not be reached.

The interpretation of the present findings is supported by the concordance between PP and ITT analyses. This agreement argues against a substantial bias introduced by differential data inclusion and suggests that the absence of a detectable treatment effect is unlikely to be attributable to the analytical approach itself. However, the study is constrained by a considerable proportion of missing data, with only 22 of 36 enrolled participants providing complete longitudinal datasets. In combination with the small sample size inherent to a pilot study, this limitation likely resulted in limited statistical power to detect small to moderate treatment effects.

No statistically significant disparities in dropout rates were observed in either study conditions. Furthermore dropout rates for the MCT plus intermittent fasting condition were comparable to previous findings regarding MCT interventions. Following these promising first results, future ITT studies should follow. A further methodological consideration is the potential for sequence and period effects inherent to the crossover design. Although participants were assigned 1:1 to treatment sequences using deterministic alternating allocation, this approach may not fully account for imbalances in baseline characteristics across sequences in small pilot samples. However, the crossover design itself partially mitigates interindividual variability by allowing participants to serve as their own controls. Future studies with larger sample sizes may benefit from stratified or block randomization of treatment sequences to further minimize the risk of sequence‐related bias and to ensure optimal balance across study periods. Seizure frequency was primarily assessed using patient‐reported seizure diaries, which are prone to under‐reporting and imprecision, especially in individuals with high seizure burden or impaired consciousness. To mitigate this limitation, we conducted weekly telephone follow‐ups including caregivers where possible, and excluded three participants because of major concerns regarding study conduct and seizure documentation. The MCT dose, which was 26 g MCT/day for all participants, should also be taken into account, to test the feasibility of a diet supplemented with an easy‐to‐use, commercially available formula. Future studies might use adjusted‐individualized and weight‐adapted MCT doses with the support of a nutritionist. To increase the practicability and to reduce the burden for the patient and the dropout of patients, an easy to use, simple way of increasing fasting and MCT‐supplementation into daily life was evaluated here.

In addition, given the frequent lack of adherence to KDs in adults,^7^, ^11^, ^14^ future studies should evaluate the possibility of additive therapy with MCT alone.^21^ Additional secondary endpoints with potential effects on seizure frequency are currently under evaluation. Among these, alterations in the gastrointestinal microbiome are of particular interest based on prior evidence.^22^ A 4‐week washout period between interventions was implemented to minimize carry‐over effects^23^, ^24^; however, ongoing microbiome analyses will determine whether this duration was sufficient. The potential effects of ASMs and macronutrient composition on seizure frequency should also be considered. To enhance feasibility and adherence, a pragmatic KD variant was implemented without strict dietary restrictions during the intervention phases. The single‐center setting limits generalizability, and masking was not feasible. Although the 28‐day washout was implemented, residual carryover effects cannot be excluded. Larger, multicenter studies are needed to determine efficacy and confirm these findings.

Population‐related limitations include the predominance of female participants, such that hormonal influences cannot be excluded. Furthermore, most participants had focal epilepsy, and potential effects in generalized epilepsy might therefore be underrepresented. Among patients with focal epilepsy, some had undergone epilepsy surgery without achieving seizure freedom (see Table 1). In such cases, safe and effective alternatives to pharmacotherapy are warranted, including ketogenic dietary approaches.^25^ Ketogenic intermittent fasting may therefore represent a feasible option for daily practice.

Overall, this crossover pilot trial suggests a signal favoring IF–MCT, with earlier and more frequent responder outcomes and seizure freedom observed only during IF–MCT. Of note, IF–MCT demonstrated a favorable safety profile and was well tolerated, supporting its potential as a clinically relevant nutritional strategy to reduce seizure burden in patients with difficult‐to‐treat epilepsy. These findings, although exploratory, support adequately powered multicenter trials to confirm efficacy, address attrition and carryover, and identify biomarkers that best capture treatment response.

## AUTHOR CONTRIBUTIONS

Wiebke Hahn: research project: conception, organization, execution; manuscript: writing. Emily Falk: data acquisition, review and critique. Ayla Balzter: data acquisition, review and critique. Tobias Foer: data acquisition, review and critique. Shanin Maierhofer: data acquisition, review and critique. Sophia Jaumann: data acquisition, review and critique. Sascha Strehlau: data analysis, review and critique. Leona Möller: review and critique. Panagiota‐Eleni Tsalouchidou: review and critique. Katja Menzler: review and critique. Lars Timmermann: review and critique. Susanne Knake: Research project: conception, organization, supervision; manuscript: review and critique.

## FUNDING INFORMATION

Unrestricted donation from Kanso to the Otto Loewi Foundation (non‐profit association supporting research, therapy, and training in neurology).

## CONFLICT OF INTEREST STATEMENT

Wiebke Hahn has received research grant from the Otto‐Loewi foundation as well as speakers’ honoraria from Kanso, both of which are unrelated to the presented work. Panagiota‐Eleni Tsalouchidou has received research funding from the German Society for Epileptology (Otfrid‐Foerster Stipendium, Deutsche Gesellschaft für Epileptologie [DGfE]), travel grants from UCB and Angelini, and honoraria for lectures from UCB, Angelini, and SWIXX BioPharma. These relationships are unrelated to the present work. Susanne Knake received speakers´ honoraria from Angelini Pharma, Bial, Eisai, Jazz Pharma, Kanso, Merck Serono, and UCB. These relationships are unrelated to the present work. The other authors declare no conflict of interest.

## CONSENT

All participants provided written informed consent before enrollment.

## CLINICAL TRIAL REGISTRATION

The trial was registered with ClinicalTrials.gov (NCT06013761).

## ETHICAL PUBLICATION STATEMENT

We confirm that we have read the Journal's position on issues involved in ethical publication and affirm that this report is consistent with those guidelines. The protocol was approved by the local ethics committee (23–92 BO).

## Supporting information


Table S1.
Table S2a.
Table S2b.
Table S3a.
Table S3b.
Table S4a.
Table S4b.
Table S4c.
Table S5.
Table S6.

## Data Availability

The data that support the findings of this study are available from the corresponding author upon reasonable request.
