# Supplementary material for: Intermittent fasting with medium‐chain triglycerides in drug‐resistant epilepsy: A pilot crossover trial
Source: Epilepsia. 2026 Jun 26;67(9):4618–30. doi: 10.1002/epi.70343 (PMC13592486; doi:10.1002/epi.70343)
Supplement: Supplementary file 1 [file EPI-67-4618-s001.docx]

**Intermittent Fasting with Medium-Chain Triglycerides in Drug-Resistant Epilepsy: A Pilot Crossover Trial**

**Supplementary Material**

Tab. S1: Seizure frequency depending on start condition

|  | baseline | start condition  4 weeks | start condition  8 weeks | start condition 12 weeks |
| --- | --- | --- | --- | --- |
| Mann-Whitney-U-Test | 32.500 | 32.500 | 42.000 | 35.500 |
| Z | -1.743 | -1.824 | -1.068 | -1.245 |
| Exact Sig. | .083 | .068 | .315 | .237 |

Tab. S2a: Correlation analyses of seizure frequency with BHB, C8 and C10 concentrations (n=22)

|  | | | **sz sum baseline** | **C8**  **baseline**  **IF MCT** | **C10**  **baseline**  **IF MCT** | **BHB baseline**  **IF MCT** |  |
| --- | --- | --- | --- | --- | --- | --- | --- |
| Spearman's rho | sz sum baseline | Correlation Coefficient | 1.000 | -.493^*^ | -0.129 | 0.390 |  |
|  |  | Sig. (2-tailed) |  | 0.020 | 0.567 | 0.072 |  |
|  |  | N | 22 | 22 | 22 | 22 |  |
|  | C8 baseline IF MCT | Correlation Coefficient | -.493^*^ | 1.000 | .509^*^ | -0.334 |  |
|  |  | Sig. (2-tailed) | 0.020 |  | 0.016 | 0.128 |  |
|  |  | N | 22 | 22 | 22 | 22 |  |
|  | C10 baseline IF MCT | Correlation Coefficient | -0.129 | .509^*^ | 1.000 | -0.021 |  |
|  |  | Sig. (2-tailed) | 0.567 | 0.016 |  | 0.924 |  |
|  |  | N | 22 | 22 | 22 | 22 |  |
|  | BHB baseline IF MCT | Correlation Coefficient | 0.390 | -0.334 | -0.021 | 1.000 |  |
|  |  | Sig. (2-tailed) | 0.072 | 0.128 | 0.924 |  |  |
|  |  | N | 22 | 22 | 22 | 22 |  |
|  | | | **sum**  **IF MCT**  **4 weeks** | **C8**  **4 weeks**  **IF MCT** | **C10**  **4 weeks**  **IF MCT** | **BHB**  **4 weeks**  **IF MCT** |  |
| Spearman's rho | sum IF MCT 4 weeks | Correlation Coefficient | 1.000 | -0.006 | 0.051 | 0.172 |  |
|  |  | Sig. (2-tailed) |  | 0.980 | 0.825 | 0.444 |  |
|  |  | N | 22 | 21 | 21 | 22 |  |
|  | C8 4 weeks IF MCT | Correlation Coefficient | -0.006 | 1.000 | .940^**^ | .470^*^ |  |
|  |  | Sig. (2-tailed) | 0.980 |  | 0.000 | 0.032 |  |
|  |  | N | 21 | 21 | 21 | 21 |  |
|  | C10 4 weeks IF MCT | Correlation Coefficient | 0.051 | .940^**^ | 1.000 | .581^**^ |  |
|  |  | Sig. (2-tailed) | 0.825 | 0.000 |  | 0.006 |  |
|  |  | N | 21 | 21 | 21 | 21 |  |
|  | BHB 4 weeks IF MCT | Correlation Coefficient | 0.172 | .470^*^ | .581^**^ | 1.000 |  |
|  |  | Sig. (2-tailed) | 0.444 | 0.032 | 0.006 |  |  |
|  |  | N | 22 | 21 | 21 | 22 |  |
|  | | | **sum sz**  **IF MCT**  **12 weeks** | **C8**  **12 weeks**  **IF MCT** | **C10**  **12 weeks**  **IF MCT** | **BHB**  **12 weeks**  **IF MCT** |  |
| Spearman's rho | sum sz IF MCT 12 weeks | Correlation Coefficient | 1.000 | 0.146 | 0.260 | 0.291 |  |
|  |  | Sig. (2-tailed) |  | 0.527 | 0.255 | 0.188 |  |
|  |  | N | 22 | 21 | 21 | 22 |  |
|  | C8 12 weeks IF MCT | Correlation Coefficient | 0.146 | 1.000 | .935^**^ | 0.390 |  |
|  |  | Sig. (2-tailed) | 0.527 |  | 0.000 | 0.081 |  |
|  |  | N | 21 | 21 | 21 | 21 |  |
|  | C10 12 weeks IF MCT | Correlation Coefficient | 0.260 | .935^**^ | 1.000 | .448^*^ |  |
|  |  | Sig. (2-tailed) | 0.255 | 0.000 |  | 0.042 |  |
|  |  | N | 21 | 21 | 21 | 21 |  |
|  | BHB 12 weeks IF MCT | Correlation Coefficient | 0.291 | 0.390 | .448^*^ | 1.000 |  |
|  |  | Sig. (2-tailed) | 0.188 | 0.081 | 0.042 |  |  |
|  |  | N | 22 | 21 | 21 | 22 |  |
|  | | | **sz sum baseline** | **C8**  **baseline**  **IF** | **C10 baseline**  **IF** | **BHB baseline**  **IF** |  |
| Spearman's rho | sz sum baseline | Correlation Coefficient | 1.000 | -0.105 | 0.125 | 0.105 |  |
|  |  | Sig. (2-tailed) |  | 0.643 | 0.579 | 0.651 |  |
|  |  | N | 22 | 22 | 22 | 21 |  |
|  | C8 baseline IF | Correlation Coefficient | -0.105 | 1.000 | .487^*^ | -0.432 |  |
|  |  | Sig. (2-tailed) | 0.643 |  | 0.021 | 0.050 |  |
|  |  | N | 22 | 22 | 22 | 21 |  |
|  | C10 baseline IF | Correlation Coefficient | 0.125 | .487^*^ | 1.000 | 0.081 |  |
|  |  | Sig. (2-tailed) | 0.579 | 0.021 |  | 0.729 |  |
|  |  | N | 22 | 22 | 22 | 21 |  |
|  | BHB baseline IF | Correlation Coefficient | 0.105 | -0.432 | 0.081 | 1.000 |  |
|  |  | Sig. (2-tailed) | 0.651 | 0.050 | 0.729 |  |  |
|  |  | N | 21 | 21 | 21 | 21 |  |
|  | | | **sum sz**  **IF**  **4 weeks** | **C8**  **4 weeks**  **IF** | **C10**  **4 weeks**  **IF** | **BHB**  **4 weeks**  **IF** |  |
| Spearman's rho | sum sz IF 4 weeks | Correlation Coefficient | 1.000 | -0.239 | -0.034 | 0.354 |  |
|  |  | Sig. (2-tailed) |  | 0.297 | 0.884 | 0.106 |  |
|  |  | N | 22 | 21 | 21 | 22 |  |
|  | C8 4 weeks IF | Correlation Coefficient | -0.239 | 1.000 | .449^*^ | -.640^**^ |  |
|  |  | Sig. (2-tailed) | 0.297 |  | 0.041 | 0.002 |  |
|  |  | N | 21 | 21 | 21 | 21 |  |
|  | C10 4 weeks IF | Correlation Coefficient | -0.034 | .449^*^ | 1.000 | -0.035 |  |
|  |  | Sig. (2-tailed) | 0.884 | 0.041 |  | 0.880 |  |
|  |  | N | 21 | 21 | 21 | 21 |  |
|  | BHB 4 weeks IF | Correlation Coefficient | 0.354 | -.640^**^ | -0.035 | 1.000 |  |
|  |  | Sig. (2-tailed) | 0.106 | 0.002 | 0.880 |  |  |
|  |  | N | 22 | 21 | 21 | 22 |  |
|  | | | **sum sz**  **IF**  **12 weeks** | **C8**  **12 weeks IF** | **C10**  **12 weeks IF** | **BHB**  **12 weeks IF** |  |
| Spearman's rho | sum sz IF 12 weeks | Correlation Coefficient | 1.000 | -0.379 | -0.003 | 0.214 |  |
|  |  | Sig. (2-tailed) |  | 0.082 | 0.990 | 0.339 |  |
|  |  | N | 22 | 22 | 22 | 22 |  |
|  | C8 12 weeks IF | Correlation Coefficient | -0.379 | 1.000 | 0.359 | -0.171 |  |
|  |  | Sig. (2-tailed) | 0.082 |  | 0.101 | 0.448 |  |
|  |  | N | 22 | 22 | 22 | 22 |  |
|  | C10 12 weeks IF | Correlation Coefficient | -0.003 | 0.359 | 1.000 | 0.384 |  |
|  |  | Sig. (2-tailed) | 0.990 | 0.101 |  | 0.078 |  |
|  |  | N | 22 | 22 | 22 | 22 |  |
|  | BHB 12 weeks IF | Correlation Coefficient | 0.214 | -0.171 | 0.384 | 1.000 |  |
|  |  | Sig. (2-tailed) | 0.339 | 0.448 | 0.078 |  |  |
|  |  | N | 22 | 22 | 22 | 22 |  |
| **. Correlation is significant at the 0.01 level (2-tailed). | | | | | | | |
| *. Correlation is significant at the 0.05 level (2-tailed). | | | | | | | |

Tab. S2b: Correlation analyses of seizure reduction of 50% or greater with BHB, C8 and C10 concentrations (n=8)

|  | | | | **sz**  **sum baseline** | | **C8**  **baseline**  **IF MCT** | | **C10 baseline**  **IF MCT** | | **BHB baseline**  **IF MCT** | |
| --- | --- | --- | --- | --- | --- | --- | --- | --- | --- | --- | --- |
| Spearman's rho | sz sum baseline | Correlation Coefficient | 1.000 | | 0.217 | | 0.096 | | 0.675 | |  |
|  |  | Sig. (2-tailed) |  | | 0.606 | | 0.820 | | 0.066 | |  |
|  |  | N | 8 | | 8 | | 8 | | 8 | |  |
|  | C8 baseline IF MCT | Correlation Coefficient | 0.217 | | 1.000 | | .810^*^ | | -0.262 | |  |
|  |  | Sig. (2-tailed) | 0.606 | |  | | 0.015 | | 0.531 | |  |
|  |  | N | 8 | | 8 | | 8 | | 8 | |  |
|  | C10 baseline IF MCT | Correlation Coefficient | 0.096 | | .810^*^ | | 1.000 | | -0.095 | |  |
|  |  | Sig. (2-tailed) | 0.820 | | 0.015 | |  | | 0.823 | |  |
|  |  | N | 8 | | 8 | | 8 | | 8 | |  |
|  | BHB baseline IF MCT | Correlation Coefficient | 0.675 | | -0.262 | | -0.095 | | 1.000 | |  |
|  |  | Sig. (2-tailed) | 0.066 | | 0.531 | | 0.823 | |  | |  |
|  |  | N | 8 | | 8 | | 8 | | 8 | |  |
|  | | | | **sum**  **IF MCT**  **4 weeks** | | **C8**  **4 weeks**  **IF MCT** | | **C10**  **4 weeks**  **IF MCT** | | **BHB**  **4 weeks**  **IF MCT** | |
| Spearman's rho | sum IF MCT 4 weeks | Correlation Coefficient | 1.000 | | 0.727 | | 0.727 | | 0.193 | |  |
|  |  | Sig. (2-tailed) |  | | 0.064 | | 0.064 | | 0.647 | |  |
|  |  | N | 8 | | 7 | | 7 | | 8 | |  |
|  | C8 4 weeks IF MCT | Correlation Coefficient | 0.727 | | 1.000 | | 1.000^**^ | | 0.107 | |  |
|  |  | Sig. (2-tailed) | 0.064 | |  | |  | | 0.819 | |  |
|  |  | N | 7 | | 7 | | 7 | | 7 | |  |
|  | C10 4 weeks IF MCT | Correlation Coefficient | 0.727 | | 1.000^**^ | | 1.000 | | 0.107 | |  |
|  |  | Sig. (2-tailed) | 0.064 | |  | |  | | 0.819 | |  |
|  |  | N | 7 | | 7 | | 7 | | 7 | |  |
|  | BHB 4 weeks IF MCT | Correlation Coefficient | 0.193 | | 0.107 | | 0.107 | | 1.000 | |  |
|  |  | Sig. (2-tailed) | 0.647 | | 0.819 | | 0.819 | |  | |  |
|  |  | N | 8 | | 7 | | 7 | | 8 | |  |
|  | | | | **sum sz**  **IF MCT**  **12 weeks** | | **C8**  **12 weeks**  **IF MCT** | | **C10**  **12 weeks**  **IF MCT** | | **BHB**  **12 weeks**  **IF MCT** | |
| Spearman's rho | sum sz IF MCT 12 weeks | Correlation Coefficient | 1.000 | | 0.750 | | 0.750 | | .755^*^ | |  |
|  |  | Sig. (2-tailed) |  | | 0.052 | | 0.052 | | 0.031 | |  |
|  |  | N | 8 | | 7 | | 7 | | 8 | |  |
|  | C8 12 weeks IF MCT | Correlation Coefficient | 0.750 | | 1.000 | | .929^**^ | | 0.429 | |  |
|  |  | Sig. (2-tailed) | 0.052 | |  | | 0.003 | | 0.337 | |  |
|  |  | N | 7 | | 7 | | 7 | | 7 | |  |
|  | C10 12 weeks IF MCT | Correlation Coefficient | 0.750 | | .929^**^ | | 1.000 | | 0.607 | |  |
|  |  | Sig. (2-tailed) | 0.052 | | 0.003 | |  | | 0.148 | |  |
|  |  | N | 7 | | 7 | | 7 | | 7 | |  |
|  | BHB 12 weeks IF MCT | Correlation Coefficient | .755^*^ | | 0.429 | | 0.607 | | 1.000 | |  |
|  |  | Sig. (2-tailed) | 0.031 | | 0.337 | | 0.148 | |  | |  |
|  |  | N | 8 | | 7 | | 7 | | 8 | |  |
|  | | | | **sz sum baseline** | | **C8**  **baseline**  **IF** | | **C10 baseline**  **IF** | | **BHB baseline**  **IF** | |
| Spearman's rho | sz sum baseline | Correlation Coefficient | 1.000 | | 0.651 | | 0.578 | | -0.313 | |  |
|  |  | Sig. (2-tailed) |  | | 0.081 | | 0.133 | | 0.450 | |  |
|  |  | N | 8 | | 8 | | 8 | | 8 | |  |
|  | C8 baseline IF | Correlation Coefficient | 0.651 | | 1.000 | | .905^**^ | | -0.524 | |  |
|  |  | Sig. (2-tailed) | 0.081 | |  | | 0.002 | | 0.183 | |  |
|  |  | N | 8 | | 8 | | 8 | | 8 | |  |
|  | C10 baseline IF | Correlation Coefficient | 0.578 | | .905^**^ | | 1.000 | | -0.571 | |  |
|  |  | Sig. (2-tailed) | 0.133 | | 0.002 | |  | | 0.139 | |  |
|  |  | N | 8 | | 8 | | 8 | | 8 | |  |
|  | BHB baseline IF | Correlation Coefficient | -0.313 | | -0.524 | | -0.571 | | 1.000 | |  |
|  |  | Sig. (2-tailed) | 0.450 | | 0.183 | | 0.139 | |  | |  |
|  |  | N | 8 | | 8 | | 8 | | 8 | |  |
|  | | | | **sum sz**  **IF**  **4 weeks** | | **C8**  **4 weeks**  **IF** | | **C10**  **4 weeks**  **IF** | | **BHB**  **4 weeks**  **IF** | |
| Spearman's rho | sum sz IF 4 weeks | Correlation Coefficient | 1.000 | | -0.432 | | -0.450 | | 0.491 | |  |
|  |  | Sig. (2-tailed) |  | | 0.333 | | 0.310 | | 0.217 | |  |
|  |  | N | 8 | | 7 | | 7 | | 8 | |  |
|  | C8 4 weeks IF | Correlation Coefficient | -0.432 | | 1.000 | | 0.607 | | -.786^*^ | |  |
|  |  | Sig. (2-tailed) | 0.333 | |  | | 0.148 | | 0.036 | |  |
|  |  | N | 7 | | 7 | | 7 | | 7 | |  |
|  | C10 4 weeks IF | Correlation Coefficient | -0.450 | | 0.607 | | 1.000 | | -0.607 | |  |
|  |  | Sig. (2-tailed) | 0.310 | | 0.148 | |  | | 0.148 | |  |
|  |  | N | 7 | | 7 | | 7 | | 7 | |  |
|  | BHB 4 weeks IF | Correlation Coefficient | 0.491 | | -.786^*^ | | -0.607 | | 1.000 | |  |
|  |  | Sig. (2-tailed) | 0.217 | | 0.036 | | 0.148 | |  | |  |
|  |  | N | 8 | | 7 | | 7 | | 8 | |  |
|  | | | | **sum sz**  **IF**  **12 weeks** | | **C8**  **12 weeks**  **IF** | | **C10**  **12 weeks**  **IF** | | **BHB**  **12 weeks**  **IF** | |
| Spearman's rho | sum sz IF 12 weeks | Correlation Coefficient | 1.000 | | -0.609 | | -0.266 | | 0.406 | |  |
|  |  | Sig. (2-tailed) |  | | 0.109 | | 0.524 | | 0.318 | |  |
|  |  | N | 8 | | 8 | | 8 | | 8 | |  |
|  | C8 12 weeks IF | Correlation Coefficient | -0.609 | | 1.000 | | 0.381 | | -0.143 | |  |
|  |  | Sig. (2-tailed) | 0.109 | |  | | 0.352 | | 0.736 | |  |
|  |  | N | 8 | | 8 | | 8 | | 8 | |  |
|  | C10 12 weeks IF | Correlation Coefficient | -0.266 | | 0.381 | | 1.000 | | 0.548 | |  |
|  |  | Sig. (2-tailed) | 0.524 | | 0.352 | |  | | 0.160 | |  |
|  |  | N | 8 | | 8 | | 8 | | 8 | |  |
|  | BHB 12 weeks IF | Correlation Coefficient | 0.406 | | -0.143 | | 0.548 | | 1.000 | |  |
|  |  | Sig. (2-tailed) | 0.318 | | 0.736 | | 0.160 | |  | |  |
|  |  | N | 8 | | 8 | | 8 | | 8 | |  |

Tab. S3a:Responders to IF MCT (defined as ≥50% reduction relative to baseline):

| **Patient** | **sz sum baseline 4 weeks** | **sz sum IF MCT 4 weeks** | **sz sum IF MCT 8 weeks** | **sz sum IF MCT 12 weeks** | **reduction at last time point** |
| --- | --- | --- | --- | --- | --- |
| 3 | 3 | 0 | 1 | 0 | 100% |
| 5 | 8 | 5 | 0 | 1 | 87.5% |
| 17 | 7 | 2 | 2 | 3 | 57.1% |
| 19 | 8 | 6 | 4 | 12 | 50% increase |
| 33 | 3 | 3 | 1 | 0 | 100% |

Tab. S3b:Responders to IF (defined as ≥50% reduction relative to baseline):

| **Patient** | **sz sum baseline 4 weeks** | **sz sum IF 4 weeks** | **sz sum sz IF 8 weeks** | **sz sum IF 12 weeks** | **reduction at last time point** |
| --- | --- | --- | --- | --- | --- |
| 16 | 58 | 41 | 32 | 16 | 72% |
| 20 | 4 | 3 | 0 | 2 | 50% |
| 26 | 6 | 9 | 0 | 10 | 66% increase |

Tab. S4a: Seizure frequency of all participants (n=36)

| **Pb** | **sz sum baseline** | **sum IF MCT 4 weeks** | **sum sz IF MCT 8 weeks** | **sum sz IF MCT 12 weeks** | **sum sz pause** | **sum sz IF 4 weeks** | **sum sz IF 8 weeks** | **sum sz IF 12 weeks** |
| --- | --- | --- | --- | --- | --- | --- | --- | --- |
| 1 | 11 | . | . | . | . | . | . | . |
| 2 | 10 | . | . | . | . | . | . | . |
| 3 | 3 | 0 | 1 | 0 | 1 | 0 | 0 | 0 |
| 4 | 8 | 11 | 9 | 10 | 11 | 8 | 10 | 11 |
| 5 | 8 | 5 | 0 | 1 | 10 | 5 | 6 | 2 |
| 6 | 420 | 345 | 300 | 340 | 275 | 285 | 325 | 320 |
| 7 | 12 | 11 | 12 | 10 | 12 | 9 | 11 | 9 |
| 8 | 20 | 15 | 19 | 17 | 10 | 17 | 14 | 12 |
| 9 | 5 | 15 | 16 | 25 | 25 | 28 | 15 | 19 |
| 10 | 3 | . | . | . | . | . | . | . |
| 11 | 3 | 2 | 2 | 2 | . | . | . | . |
| 12 | 15 | . | . | . | . | . | . | . |
| 13 | 8 | 5 | 8 | 5 | 5 | 4 | 3 | 4 |
| 14 | 3 | . | . | . | 2 | 1 | 2 | 1 |
| 15 | 8 | 31 | 36 | 23 | 25 | 29 | 19 | 16 |
| 16 | 58 | 20 | 10 | 10 | 12 | 41 | 32 | 16 |
| 17 | 7 | 2 | 2 | 3 | 1 | 2 | 1 | 2 |
| 18 | 11 | 8 | 12 | 11 | 12 | 9 | 6 | 8 |
| 19 | 8 | 6 | 4 | 12 | 7 | 7 | 5 | 8 |
| 20 | 4 | 2 | 0 | 2 | 5 | 3 | 0 | 2 |
| 21 | 7 | 11 | 7 | 9 | 6 | 3 | 8 | 4 |
| 22 | 56 | 35 | . | . | 34 | 38 | 24 | 24 |
| 23 | 50 | 48 | . | . | . | . | . | . |
| 24 | 12 | . | . | . | . | . | . | . |
| 25 | 5 | . | . | . | . | . | . | . |
| 26 | 6 | 3 | 2 | 6 | 2 | 9 | 0 | 10 |
| 27 | 43 | 31 | . | . | . | . | . | . |
| 28 | 94 | 216 | 196 | 131 | 157 | 170 | 255 | 185 |
| 29 | 3 | . | . | . | . | . | . | . |
| 30 | 12 | 9 | 13 | 20 | 10 | 13 | 10 | 7 |
| 31 | 9 | 12 | 15 | 20 | 17 | 28 | . | . |
| 32 | 51 | 41 | 52 | 43 | 44 | 50 | 42 | 39 |
| 33 | 3 | 3 | 1 | 0 | 1 | 0 | 1 | 2 |
| 34 | 3 | 5 | 5 | 3 | 3 | 8 | 5 | 5 |
| 35 | 10 | 12 | 7 | 6 | 7 | 14 | 9 | 7 |
| 36 | 3 | 3 | 3 | 3 | 3 | 3 | 3 | 3 |

Yellow mark indicates patients who had to be excluded. Orange mark indicates dropouts “.” indicates missing values. Pb= proband; sz= seizure frequency; IF MCT= intermittent fasting+ MCT intake; IF= intermittent fasting.

Tab. S4b: Intention to treat-analysis of seizure frequency for all study participants across all eight time points in a within-subject-design (Friedman test)

| N | 23 |
| --- | --- |
| Chi-Square | 9.925 |
| df | 7 |
| Asymp. Sign | .193 |

Tab.S4c: Intention to treat-analysis of seizure frequency of all study participants depending on the baseline condition (IF MCT vs. IF) at the individual time points in pairwise comparison (Mann–Whitney U test)

|  | baseline | start condition  4 weeks | start condition  8 weeks | start condition 12 weeks |
| --- | --- | --- | --- | --- |
| Mann-Whitney-U-Test | 115.0 | 85.000 | 72.500 | 67.500 |
| Z | -1.498 | -1.142 | -1.154 | -1.384 |
| Exact Sig. | .143 | .267 | .254 | .170 |

Tab. S5: CONSORT checklist

| **Section/topic** | **No.** | **CONSORT checklist item description** | **Report** | **Manuscript location** |
| --- | --- | --- | --- | --- |
| **Title and abstract** |  |  |  |  |
| Title and structured abstract |  |  |  |  |
|  | 1a | Identified as a randomized crossover trial comparing IF16:8 vs IF 16:8+MCT in adults with drug-resistant epilepsy | yes | title page, abstract |
|  | 1b | Therefore, see abstract section: The abstract includes design, setting, participants, interventions, primary outcome, results, trial registration. | yes | abstract |
| **Open science** |  |  |  |  |
| Trial registration | 2 | Clinical trials.gov ID: NCT06013761; first submitted: 2023-08-08; first posted: 2023-08-28. URL: https://clinicaltrials.gov/study/NCT06013761?tab=researcher | yes | abstract, supplementary material (original protocol) |
| Protocol and statistical analysis plan | 3 | Online available at Clinicaltrials.gov: https://clinicaltrials.gov/study/NCT06013761?tab=researcher#trial-description; also study plan (original protocol; additional material) | yes | abstract, supplementary material (original protocol) |
| Data sharing | 4 | The study data will be made available upon reasonable request. | yes | title page |
| Funding and conflicts of interest |  |  |  |  |
|  | 5a | Unrestricted donation from Kanso to the Otto Loewi Foundation (a non-profit association supporting research, therapy, and training in neurology). The funder of the study had no role in study design, data collection, data analysis, data interpretation, or writing of the report. | yes | title page |
|  | 5b | All grants of the authors are unrelated to the presented work. | yes | title page |
| **Introduction** |  |  |  |  |
| Background and rationale | 6 | To evaluate whether 16:8 intermittent fasting with exogenous MCT supplementation improves seizure outcomes in adults with drug-resistant epilepsy. | yes | abstract, introduction |
| **Objectives** |  |  |  |  |
| Objectives | 7 | IF–MCT demonstrated a favourable safety profile and was well tolerated, supporting its potential as a clinically relevant nutritional strategy to reduce seizure burden in patients with difficult-to-treat epilepsy | yes | discussion |
| **Methods** |  |  |  |  |
| Patient and public involvement | 8 | Not available. | no |  |
| Trial design | 9 | Randomized, open-label, crossover assignment. | yes | abstract, study procedure |
| Changes to trial protocol | 10 | Pilot target n=28, actual enrollment n=22; rationale based on feasibility. | yes | statistical analysis, discussion |
| Trial setting | 11 | Single-center epilepsy center at Philipps-University Marburg, Germany. | yes | abstract, study design |
| Eligibility criteria |  |  |  |  |
|  | 12 | Inclusion criteria: - subjects able to provide informed consent - drug-resistant epilepsy - at least 3 seizures per month Exclusion Criteria: - pregnancy - breast feeding period - metabolic disorder (e.g. diabetes, liver cirrhosis, kidney disease) - eating Disorder (e.g. anorexia, bulimia) - chronic inflammatory gut disease - active cancerous disease - antibiotics within the last 3 months | yes | participants |
|  | 12b | Not applicable. | no |  |
| Intervention and comparator | 13 | Participants completed two 12-week intervention periods— (1) 16:8 intermittent fasting plus MCT supplementation (IF–MCT) and (2) 16:8 intermittent fasting alone (IF)—separated by a four-week washout period | yes | original protocol |
| Outcomes | 14 | Primary outcome: seizure frequency during adjunct 16:8 intermittent fasting, with or without exogenous MCT supplementation, compared with standard pharmacotherapy alone (baseline), in adults with DRE. Secondary outcomes (eg, neuronal connectivity and neuropsychological outcomes) will be reported separately. | yes | outcomes, details for secondary outcomes in supplementary material (original protocol) |
| Harms | 15 | Not defined because not expected. | no |  |
| Sample size | 16 | Planned sample size: 28. Analyzed (due to dropouts or exclusion): 22 | yes | abstract, participants |
| **Randomization** |  |  |  |  |
| Sequence generation |  |  |  |  |
|  | 17a | Wiebke Hahn, who was one of the principal investigators of the study. | yes | randomisation and masking |
|  | 17b | Deterministic alternating allocation. | yes | abstract |
| Allocation concealment mechanism | 18 | Participants were allocated (1:1) to the initial intervention sequence using a deterministic alternating scheme based on enrolment order and study identification number. Participants with odd identification numbers started with IF–MCT and those with even identification numbers started with IF. | yes | randomisation and masking |
| Implementation | 19 |  |  |  |
| Blinding |  | Only the principal investigators (W. Hahn and S. Knake) had access to the allocation sequence. | yes | original protocol |
|  | 20 | None. Owing to the nature of the dietary interventions, participants and study staff were not masked to treatment assignment. | yes | randomisation and masking |
|  |  |  |  |  |
| Statistical methods |  |  |  |  |
|  | 21a | Within-participant comparisons were done using the Friedman test. Between-intervention comparisons were done using the Mann–Whitney U test. Spearman rank correlation was used for correlation analyses, and post-hoc comparisons were done using the Wilcoxon signed-rank test. A Bonferroni correction was applied to control for type I error due to multiple comparisons. Statistical analyses were performed using SPSS version 30 (IBM Corp., 2024). | yes | statistical analysis |
|  | 21b | Analyses were performed in the per-protocol population, defined as participants who completed both intervention periods according to the protocol. Missing primary outcome data were handled by complete-case analysis, and no imputation was performed. | yes | statistical analysis |
|  | 21c | Missing primary outcome data were handled by complete-case analysis, and no imputation was performed. | yes | statistical analysis |
|  | 21d | Post-hoc comparisons were done using the Wilcoxon signed-rank test. A Bonferroni correction was applied to control for type I error due to multiple comparisons. | yes | statistical analysis |
| **Results** |  |  |  |  |
| Participant flow, including flow diagram |  |  |  |  |
|  | 22a | See figure 1 (study design flow chart) and CONSORT flow diagram | yes | supplementary material: CONSORT flow diagram |
|  | 22b | Three participants withdrew consent before the intervention. Eight discontinued the intervention: two because of gastrointestinal adverse events, two because of time constraints, one because study materials were lost, two because of recurrence of depressive symptoms unrelated to study procedures, and one for unspecified reasons. Of the 25 participants who completed the trial, three were excluded from the analysis owing to substantial concerns about adherence to study procedures (figure 1). | yes | participants; fig. 1 |
| Recruitment |  |  |  |  |
|  | 23a | August 1, 2023 - May 31, 2025 | yes | study design |
|  | 23b | May 31, 2025 | yes | study design |
| Intervention and comparator delivery |  |  |  |  |
|  | 24a | Crossover-design; each participant received each condition. Seizures (including auras) were recorded using a standardised daily seizure diary, and adherence to fasting and eating windows was monitored with daily food logs. Data is available upon reasonable request. | yes | outcomes, data sharin (title page) |
|  | 24b | Participants attended seven on-site visits. Weekly telephone contacts were used to support adherence. | yes | procedures |
| Baseline data | 25 | Therefore, see table 1: Patient characteristics | yes | table 1 |
| Numbers analyzed, outcomes, and estimation | 26 | Online complete case-analysis for the primary outcome. For results, see result section. | yes |  |
| Harms | 27 | Patients who discontinued due to adverse effects: gastrointestinal adverse events (2), recurrence of depressive symptoms unrelated to study procedures (2). | yes | participants |
| Ancillary analyses | 28 | Therefore, see statistical analysis and results: Within-participant comparisons were done using the Friedman test. Between-intervention comparisons were done using the Mann–Whitney U test. Spearman rank correlation was used for correlation analyses, and post-hoc comparisons were done using the Wilcoxon signed-rank test. | yes | statistical analysis, results |
| **Discussion** |  |  |  |  |
| Interpretation | 29 | Therefore, the discussion section. | yes | discussion |
| Limitations | 30 | Therefore, see methodological and population-related limitations in the discussion section. | yes | discussion |

Tab. S6: Weight per patient (kg) and MCT dose (g per kg body weight) at the start and end of the study

| **patient** | **weight baseline (kg)** | **weight T2.2 (kg)** | **MCT (g/kg)** |
| --- | --- | --- | --- |
| 3 | 57.0 | 56.7 | 0.46 |
| 4 | 66.9 | n.a | 0.39 |
| 5 | 55. 4 | 58.6 | 0.47 |
| 6 | 54.2 | 54.5 | 0.48 |
| 7 | 59.6 | 60.6 | 0.44 |
| 8 | 63.8 | 58.7 | 0.41 |
| 9 | 62.6 | n.a. | 0.42 |
| 13 | 65.5 | 60.8 | 0.40 |
| 15 | 83.7 | 83.6 | 0.31 |
| 16 | 76.1 | 73.1 | 0.34 |
| 17 | 63.5 | 60.6 | 0.41 |
| 18 | 64.5 | 65.3 | 0.40 |
| 19 | 68.0 | 71.6 | 0.38 |
| 20 | 54.3 | 53.0 | 0.48 |
| 21 | 65.4 | n.a. | 0.40 |
| 26 | 69.9 | 69.0 | 0.37 |
| 28 | 111.2 | 107.5 | 0.23 |
| 30 | 73.0 | n.a. | 0.36 |
| 32 | 74.4 | n.a | 0.35 |
| 33 | 71.8 | 73.7 | 0.36 |
| 34 | 62.8 | 63.9 | 0.41 |
| 35 | 101.0 | 98.0 | 0.26 |

## **IF-MCT 16:8: Investigating the Influence of Intermittent Fasting With and Without MCTs in Patients With Drug-resistant Epilepsy**

## **(IF-MCT16:8)**

**Original trial protocol**

Important note:

This study protocol was submitted to the Marburg Ethics Committee on 9^th^ May 2023 and approved. For manuscript publication, the study protocol was translated from German into English.

The study protocol, which was submitted to ClinicalTrials on 8^th^ August 2023 and posted there on 28^th^ August 2023 (ClinicalTrials.gov ID; NCT0601376) can be found at the following link: https://clinicaltrials.gov/study/NCT06013761#study-plan (last accessed online: 19th January, 2026).

**A - Benefits / Expected Insights of the Planned Project**

The primary aim of the planned prospective, single-center, randomized, experimental pilot study is to investigate the effect of intermittent fasting according to the 16:8 principle, in combination with a one-time additional intake of certified medium-chain MCT fats, on seizure frequency in patients with therapy-refractory epilepsy. Secondarily, the study will also examine the influence of this modified dietary approach on aspects such as quality of life, self-efficacy, and various potential biomarkers, including (among others) the composition of the gut microbiome as well as neuronal networks in patients with pharmacoresistant epilepsy.

**B- Formal information**

1. **Title: IF-MCT 16:8: Investigating the Influence of Intermittent Fasting With and Without MCTs in Patients With Drug-resistant Epilepsy (IF-MCT16:8)**
2. **Responsible Principal Investigators in Marburg**

Prof. Dr. Susanne Knake;

Dr. Wiebke Hahn

**3.** **Multicenter study**

No.

**4. Sponsor(s) (if applicable: own department/institute):**

Funding provided by the Otto Loewi Foundation (Otto-Loewi-Stiftung e.V.), a non-profit association supporting research, therapy, and education in neurology (formerly the Neurology Support Association; internal/in-house).

**5.** **Funding**

Non-public third-party funding. Name of the funding body: Otto Loewi Foundation (Otto-Loewi Stiftung e.V.), a non-profit association supporting research, therapy, and education in neurology.

Note: The study is supported by a donation to the Otto Loewi Foundation as well as by the provision of nutritional supplement shakes by company Kanso/Dr. Schär (Dr. Schär AG/SPA, Winkelau 9, 39014 Burgstall (BZ) Italy).

**6. Trial Agreement / Investigator Fee**

No.

**C Project information**

**7. Study project**

**Rationale / Study Justification**

Epilepsy is one of the most common chronic neurological disorders and is associated with far-reaching cognitive, psychological, and social consequences. Worldwide prevalence is estimated at around 50 million affected individuals. Current incidence is approximately 2.4 million patients^1^. One in three patients does not become seizure-free with conventional pharmacotherapy. According to the criteria of the International League Against Epilepsy (ILAE), pharmacoresistant epilepsy is defined as a failure to achieve seizure freedom after adequate trials of two appropriately chosen and tolerated first-line antiseizure medications (ASM) administered at sufficient doses. The likelihood of achieving seizure freedom with each additional medication is only in the single-digit percentage range.

For this reason, additive therapeutic approaches such as the ketogenic diet play an important role, particularly in pharmacoresistant epilepsies. Various studies in pediatric populations have shown a significant reduction in seizure frequency with the ketogenic diet^2^ and consequently a long-term reduction in epilepsy-related medical costs^3^. The ketogenic diet induces a metabolic fasting state in the body. This ketogenic metabolic state is achieved through a high-fat and low-carbohydrate diet. The primary goal is the endogenous production of ketone bodies in the liver, which are used instead of glucose to generate ATP as the main energy source. This metabolic shift leads to biochemical, metabolic, and hormonal changes that may reduce the severity and frequency of epileptic seizures, although the exact mechanisms are not yet fully understood^4^. Several pathophysiological pathways have been proposed through which the gut microbiome may influence seizure frequency, including the release of GABAergic neurotransmitters or precursor amino acids (e.g., tryptophan), modulation of neuronal signaling pathways—particularly via the vagus nerve—as well as a reduction in short-chain fatty acids, which may result in an anti-inflammatory effect^5^.

In the context of the ketogenic diet, several forms or variants must be distinguished: the classical ketogenic diet, the modified Atkins diet, the low glycemic index diet, and the ketogenic diet based on medium-chain triglycerides. In the classical ketogenic diet (cKD), the composition of a meal corresponds to a ratio of 4 g fat to 1 g protein and carbohydrates (a so-called ketogenic ratio of 4:1). Thus, 90% of dietary energy intake is derived from fats, which is very difficult to implement in everyday life for adult patients and is therefore often not pursued. A less restrictive alternative to the cKD is the modified Atkins diet (MAD). In this dietary approach, in which a meal consists predominantly of fats (approximately 60%), the ketogenic ratio is around 1:1. In the low glycemic index diet (LGID), each meal consists of protein, carbohydrates, and fat in a composition that is not precisely defined. Daily carbohydrate intake should be limited to 40–60 g per day, and carbohydrates with a low impact on blood glucose levels should be consumed. Another dietary approach related to ketosis is ketogenic nutrition therapy using medium-chain triglycerides (MCTs). MCTs have a higher ketogenic potential than the long-chain fatty acids used in the cKD. Therefore, larger amounts of carbohydrates and protein can be consumed within an MCT-based dietary regimen. What all of these dietary forms have in common is the need for specific preparation of each meal using detailed meal plans and often an initiation of the additive therapy in an inpatient setting or under supervision by trained staff^2^. All presented forms of ketogenic nutrition require long-term careful planning of all meals and a high level of discipline from patients and their families. Furthermore, it remains unclear what degree of ketosis must be achieved to obtain a therapeutic effect. Especially in adults, lack of adherence appears to play an important role in the effectiveness of the ketogenic diet^6^.

An alternative form of fasting that is easier to integrate into daily life is intermittent fasting, which is recognized by the German Nutrition Society (DGE) and the Federal Centre for Nutrition (BZfE). This dietary approach, also referred to as intermittent fasting (IF), is attributed with various beneficial metabolic effects, most notably weight reduction. In contrast to other fasting regimens not described here, IF is intended to be applied as a long-term dietary pattern.

In this context, the DGE and BZfE describe various dietary protocols in which eating and fasting phases alternate. These include the two-day diet, the 5:2 diet, alternate-day fasting, dinner cancelling, and the Leangains method. The two-day diet, originally developed for weight loss in breast cancer patients^7^ allows a maximum intake of 650 kilocalories on two consecutive days per week. The food consumed should be low in carbohydrates and high in protein. In the 5:2 diet, normal eating is maintained on five days of the week without restrictions regarding food composition or selection, which is intended to improve adherence. On two days per week, fasting days are implemented during which only 25% of the usual caloric intake should be consumed, primarily through vegetables, whole-grain products, and protein-rich foods. An alternative is alternate-day fasting (ADF), in which fasting and non-fasting days alternate^8^. On fasting days, only 25% of the usual energy intake should be consumed, while there are no restrictions on non-fasting days. Dinner cancelling refers to a form of IF in which dinner is omitted on selected days (at least two) per week, resulting in a fasting period of at least 14 hours. This pauses insulin production, promotes weight loss, and may improve sleep quality. Only water and calorie-free beverages such as unsweetened tea should be consumed (DGE, BZfE). Another IF method is the Leangains method, better known as the 16:8 method. In this dietary regimen, a fasting period of 16 hours is maintained. During an 8-hour window (e.g., 12:00–20:00), three meals are consumed. After the evening meal, a 16-hour fasting period follows (e.g., from 20:00 until 12:00 the next day). There are no restrictions regarding the composition of the diet. The aim of this method is to reduce insulin secretion and promote weight loss^9^.

**Current state of research**

To the best of our knowledge, there is currently only one study investigating the effect of intermittent fasting in patients with pharmacoresistant epilepsy: In 2013, Hartman and colleagues examined adherence and seizure reduction in a retrospective case series using the two-day diet in combination with MCT fats over a period of two months in six children aged 2 to 7 years with pharmacoresistant epilepsy^10^. Three participants showed good adherence to the two-day diet. Four out of six participants demonstrated a significant reduction in seizure frequency^10^. To the best of our knowledge, no data are available on the seizure-reducing effects of intermittent fasting in adult patients with pharmacoresistant epilepsy. Based on the current evidence, it remains unclear whether intermittent fasting alone, or in combination with additional MCT fat supplementation, can achieve a seizure-reducing effect in patients with epilepsy. Compared with the ketogenic diet (KD), the 16:8 intermittent fasting method has the major advantage of being easier to integrate into daily life.

Despite the long-standing use of the classical ketogenic diet as an adjunctive therapeutic approach, its exact neuroprotective mechanisms remain unclear. In a mouse model, Olson and colleagues (2018) were the first to demonstrate an association between the ketogenic diet’s impact on the gut microbiome and neuronal modulation: In contrast to mice treated with antimicrobials—who did not respond to the KD—mice fed a ketogenic diet showed an altered microbiome composition^11^. Among other findings, ketogenic-fed mice exhibited reduced alpha diversity, a measure of within-sample species richness. Alpha diversity reflects the number of different microbial species detectable within an individual in the examined region. In contrast, beta diversity represents the variability between individuals within the same habitat with regard to detected microorganisms. In addition to reduced alpha diversity, Olson and colleagues observed an increase in certain bacterial subpopulations that may be neuroprotective or seizure-suppressive (e.g., Akkermansia muciniphila and Parabacteroides), altered serum amino acid concentrations, and a reduction in bilateral tonic–clonic seizures^11,12^. In a 2021 review, Chatzikonstantinou and colleagues summarized the current evidence on the gut microbiome in pharmacoresistant epilepsy^5^: Based on available data, the microbiome of patients with pharmacoresistant epilepsy appears to differ from that of healthy control participants, particularly with regard to increased alpha diversity^13,14^. An influence of the ketogenic diet on gut microbiome alterations in pediatric epilepsy has been proposed, among others, by Tagliabue and colleagues^15^. However, evidence in adults with pharmacoresistant epilepsy is still lacking.

With regard to the gut microbiome and intermittent fasting, data are currently available only for healthy individuals. In 2022, Khan and colleagues investigated the effects of the 16:8 method over 26 days in 60 healthy Pakistani female participants, assessing BMI, serum lipid profiles, and the gut microbiome^16^. In addition to a reduction in BMI among obese participants and a decrease in LDL cholesterol levels, the authors reported changes in bacterial diversity and, in particular, an increase in bifidobacteria and lactobacilli^16^. The latter is often interpreted in the context of weight loss.

Furthermore, numerous studies indicate bidirectional effects of the (intestinal) microbiome on the T-cell–mediated innate immune system, which can be modulated in either a beneficial or detrimental manner depending on microbial subpopulations and may thereby contribute to the development of autoimmune diseases.

Beyond the effects already described, the ketogenic diet also appears to have a significant impact on the stability of neuronal networks: In an fMRI study on biomarkers of brain aging, it was shown that endogenous and exogenous ketone bodies compared with a standard glucose-based diet had an effect on functional connectivity between different brain regions in young, healthy participants^17^. The study showed that ketone bodies—regardless of how they were generated (one week of a classical ketogenic diet, fasting, or ingestion of a ketogenic drink)—remained available as cerebral energy substrates significantly longer than glucose and thus contributed to stabilization of neuronal networks^17^.

**Research Question**

What is the impact of intermittent fasting according to the 16:8 method, in combination with exogenously administered MCT fats, on seizure frequency, biomarkers, and neuronal networks in patients with pharmacoresistant epilepsy?

**8. Type of research question**

Evaluation of a treatment intervention (efficacy, safety).

**9. Study design**

Experimental randomized (interventional) study.

A simple, non-stratified randomization into two groups will be conducted, assigning participants by chance to an intervention group (= IG1) and a control group (= CG1). After a defined period in the first study phase and a defined washout period, a group switch will take place according to a cross-over design (see Table 1 and Table 2): This means that participants initially assigned to IG1 will automatically become participants in control group 2 (= CG2), and participants initially assigned to CG1 will automatically become participants in intervention group 2 (= IG2). No blinding will be implemented in this first interventional study investigating intermittent fasting with MCT supplementation in patients with pharmacoresistant epilepsy.

**10.** **Time line**

Planned study start: expected June 2023; expected study end: end of 2025.

**11.	Hypothesis testing**

- Primary hypothesis: Intermittent fasting plus once-daily intake of a dietary supplement containing MCT fats leads to a reduced seizure frequency in patients with pharmacoresistant epilepsy.
- Secondary hypothesis 1: Intermittent fasting using the 16:8 method can be well integrated into the daily lives of patients with epilepsy, thereby supporting treatment adherence.
- Secondary hypothesis 2: Intermittent fasting combined with once-daily intake of a dietary supplement containing MCT fats can achieve a sufficient level of ketosis.
- Secondary hypothesis 3: Intermittent fasting promotes patients’ self-efficacy.
- Secondary hypothesis 4: The more everyday-feasible ketogenic diet (KD) improves patients’ quality of life.
- Secondary hypothesis 5: Intermittent fasting plus once-daily intake of MCT fats instead of breakfast leads to changes in the assessed biomarkers.
- Secondary hypothesis 6: Intermittent fasting plus once-daily intake of MCT fats leads to changes in neuronal networks in patients with epilepsy, as measured by DTI and resting-state fMRI.
- Secondary hypothesis 7: Intermittent fasting plus daily intake of MCT fats leads to an altered T-cell–mediated immune response in immunological status analyses.

**12. Description of the methodological study procedure**

First, the study will be prepared and participants will be enrolled (see table 1). This will be followed by a four-week baseline assessment period. After completion of the baseline phase, study phase 1 will begin and will last for three months. Following a four-week break for both groups, a group switch will take place: intervention group 1 (IG1) will become control group 2 (CG2), and control group 1 (CG1) will become intervention group 2 (IG2). This marks the start of study phase 2, which will also last for three months. After three months in study phase 2, the study will be completed and data analysis will begin.

Fig. 1: Study overview and timeline

Preparation/ enrollment

Baseline assessment (duration 4 weeks)

Study phase 1 (duration 3 months) with IG1 and CG1

Pause (duration 4 weeks)

Study phase 2 (duration 3 months) with IG2 and CG2

End of the study and data analysis

During the preparation and enrollment phase, patients will be informed about the study and provided with detailed information. Inclusion and exclusion criteria will be assessed. Written informed consent will be obtained if patients wish to participate in the study. Participants will receive contact details in case any problems occur during study participation. In addition, patients will be provided with an information sheet about the study for their treating general practitioner as well as for their (if applicable) treating outpatient neurologist. These documents will be handed to participants personally in order to avoid any violation of data protection regulations. Participants will also receive a first stool sample collection container.

Following the preparation/enrollment phase, the baseline assessment will be conducted. This baseline period will last four weeks and will begin at the time of a visit to our university outpatient clinic. Current seizure frequency will be recorded over a period of four weeks. In addition, vital signs (blood pressure, heart rate, sPO₂, temperature) will be measured non-invasively. BMI will be assessed. A first blood sample will be taken, including a basic laboratory panel. Furthermore, participants will be asked to provide a fresh stool sample for the first microbiome analysis (either during this visit, if possible, or otherwise within the baseline period during which no intervention will yet take place). At the end of the baseline assessment, i.e., at time point T0.1 (start of study phase 1), all participants will undergo an EEG and a cerebral MRI (including DTI and resting-state imaging), as well as duplex ultrasonography of the cerebral supplying arteries (including intima–media thickness).

Study phase 1 begins with random allocation of participants to intervention group 1 (= IG1) and control group 1 (= CG1). At this point, it should be noted that a cross-over design will be applied. This means that participants initially assigned to IG1 will automatically become participants in control group 2 (= CG2), and participants initially assigned to CG1 will automatically become participants in intervention group 2 (= IG2). Both the intervention and control groups will perform intermittent fasting according to the 16:8 method for a period of three months. The intervention group will additionally take an approved, certified dietary supplement product containing MCT fats (FA Kanso: product KetoEpi) once daily in the morning at 10:00 a.m. During the same period, the control group will perform intermittent fasting only. Participants in both groups may eat as usual between 12:00 p.m. and 8:00 p.m. There will be no restrictions regarding the composition of food in order to ensure the highest possible adherence. After 8:00 p.m. and until 12:00 p.m., both groups will refrain from consuming any additional food or caloric beverages, with the exception of the specified once-daily intake of MCT fats in the respective intervention group.

All participants will keep a seizure and diet diary. All participants will document ketosis on a weekly basis. Study phase 1 will be divided into three assessment time points (T0.1, T1.1, T2.1). At all time points, participants will be asked to attend the university outpatient clinic.

**• T0.1 (after completion of baseline; unless otherwise specified; applicable to both groups):**

o Assessment of vital parameters

o Large blood sample including immunological status

o Documentation of seizure frequency using the seizure diary

o Instruction and provision of the diet diary

o Assessment of questionnaires: self-efficacy and quality of life

o Provision of a ketone meter and test strips, including instruction on measurement

o Provision of the keto shake (IG1 only)

o Cerebral MRI (cMRI)

o EEG

o Duplex ultrasonography (including intima–media thickness)

**• T1.1 (1 month after T0.1):**

o Small blood sample

o Assessment of seizure frequency

o Assessment of self-efficacy and quality of life

**• T1.2 (3 months after T0.1):**

o Large blood sample including immunological status

o Vital parameters

o BMI

o Assessment of seizure frequency

o Questionnaires: self-efficacy and quality of life

o Stool sample with microbiome analysis

o Cerebral MRI (cMRI)

o EEG

o Duplex ultrasonography (including intima–media thickness)

To reduce the risk of a carry-over effect due to intermittent fasting or intermittent fasting combined with MCT fats, a four-week break will be implemented for all participants prior to the start of study phase 2. During this period, participants should follow their usual diet. No additional documentation will be required during this time, except for documentation of seizure frequency.

Study phase 2 will begin after a four-week intermittent fasting break in order to minimize a potential influence of the dietary interventions and to prevent a carry-over effect. Participants who previously belonged to IG1 will now become CG2, and conversely, participants who previously belonged to CG1 will become IG2. This means that participants who performed intermittent fasting only during study phase 1 will now additionally take the shake during study phase 2. Participants who performed intermittent fasting and consumed the MCT shake during study phase 1 will now perform intermittent fasting only. Apart from this switch, study phase 2 will be conducted identically to study phase 1.

**• T0.2 (after completion of the break; unless otherwise specified; applicable to both groups):**

o Assessment of vital parameters

o Large blood sample including immunological status

o Documentation of seizure frequency using the seizure diary

o Instruction and provision of the diet diary

o Assessment of questionnaires: self-efficacy and quality of life

o Provision of a ketone meter and test strips, including instruction on measurement

o Provision of the keto shake (IG2 only)

o Stool sample with microbiome analysis

**• T2.1 (1 month after T0.2):**

o Small blood sample

o Assessment of seizure frequency

o Assessment of self-efficacy and quality of life

**• T2.2 (3 months after T0.2):**

o Large blood sample including immunological status

o Vital parameters

o BMI

o Assessment of seizure frequency

o Questionnaires: self-efficacy and quality of life

o Stool sample with microbiome analysis

o Cerebral MRI (cMRI)

o EEG

o Duplex ultrasonography (including intima–media thickness)

End of the Study and Data Analysis:After three months and completion of time point T2.2 in study phase 2, the study will end and the analysis phase will begin.

**13. Outcome Measures / Endpoints**

Primary outcome measure: Reduction in seizure frequency

Secondary outcome measures:

- Self-efficacy
- Quality of life
- Biomarkers (especially ketosis, microbial analysis of stool composition, and subpopulation analysis)
- Neuronal networks
- Immune response (especially T-cell–mediated innate immune response)

The clinical/intervention group (IG) will perform 16:8 intermittent fasting with a normal diet between 12:00 p.m. and 8:00 p.m., and will additionally take an approved, certified dietary supplement containing MCT fats (“Kanso KetoEpi”) once daily instead of a morning meal. The control group (CG) will perform 16:8 intermittent fasting only, with a normal diet between 12:00 p.m. and 8:00 p.m. In study phase 2, a group switch will take place, meaning that the former intervention group will become the control group and vice versa.

**14. Number of patients/participants including sample size calculation or justification (presentation of the statistical considerations underlying the sample size)**

To the best of our knowledge, there are currently no studies on intermittent fasting combined with MCT fats in adult patients with pharmacoresistant epilepsy. We are aware only of the above-mentioned case series by Hartman and colleagues (2013) involving six children with pharmacoresistant epilepsy^10^. As a result, a power calculation indicates that, in order to achieve an 80% probability at a significance level of p < 0.05, the required number of participants would be in the high three-digit range—an estimate that is very likely to substantially overestimate the actual number of participants needed. Consequently, an initial subsample of a total of 28 participants will be recruited, which will allow for a more appropriate calculation of the true required sample size to achieve acceptable statistical power for the primary hypotheses. The number of participants actually required will then be adjusted based on the newly determined, more reliable value obtained from a repeated power calculation. Recruitment will end once the required number of patients have successfully completed the study. At our epilepsy center, we treat approximately 1.000 patients with pharmacoresistant epilepsy per year. Recruitment is therefore highly feasible.

**15.** **Particularly vulnerable individuals**

Minors and adults unable to give informed consent. This is a prospective, single-center, experimental interventional study involving adult, capable-of-consent, non-pregnant patients with pharmacoresistant epilepsy and without relevant comorbidities (see exclusion criteria). As this is the first study in this field, individuals who are unable to provide informed consent will not be included.

**16. Inclusion/Exclusion Criteria for the Selection of Patients/Participants**

Inclusion criteria:

- subjects able to provide informed consent
- drug-resistant epilepsy
- at least 3 seizures per month

Exclusion Criteria:

- pregnancy
- breast feeding period
- metabolic disorder (e.g. diabetes, liver cirrhosis, kidney disease)
- eating disorder (e.g. anorexia, bulimia)
- chronic inflammatory gut disease
- active cancerous disease
- antibiotics within the last 3 months

**17. Description of the statistical analysis procedure**

As part of the study, statistical analysis will be supported and/or advised by our colleagues in neuropsychology within our research group. The calculation of the parameters of interest, descriptive presentation, and inferential statistical testing of the hypotheses will be performed using the statistical software SPSS. The analysis of imaging morphology data will be conducted using SPM software integrated into Matlab. The corresponding beta values derived from the imaging files will then be further processed in SPSS.

**Hyothesis testing studies:**

17.1 Formulation of the null hypothesis for the primary outcome measure:

Once-daily intake of a dietary supplement containing MCT fats following intermittent fasting in the intervention group (IG) leads to a reduced seizure frequency in patients with pharmacoresistant epilepsy.

17.2 Statistical test procedure for hypothesis testing

First, hypothesis testing will be performed using the statistical software SPSS to evaluate the primary hypothesis that intermittent fasting combined with exogenously administered MCT fats leads to a reduction in seizure frequency in patients with pharmacoresistant epilepsy. Subsequently, the secondary endpoints will also be tested. Parametric methods (e.g., mixed ANOVA / two-factor analysis of variance with repeated measures) will be applied. If the assumptions for these methods are not met, appropriate non-parametric procedures will be used instead. If missing data points occur frequently in certain sections, these will be excluded from the analysis.

17.3 Minimal relevant effect size and corresponding power of the test procedure

If no measurable group differences are observed in this first study in the field, assuming medium effect sizes, a post hoc power analysis will be conducted for future studies.

17.4 Conclusions to be drawn in the event that the null hypothesis is rejected

- No change in seizure frequency through an everyday-feasible ketogenic diet (with MCT fats)

- Failure to achieve sufficient ketosis through intermittent fasting with MCT fats

- Low adherence among study participants

**D Burden and risc**

**18. Description and specification of all project-related procedures involving patients or participants** (in particular, study-related measures that differ from routine clinical care and benefit–risk assessment)**:**

A randomized allocation into two groups is planned: an intervention group (IG) and a control group (CG). Due to the described cross-over design, a group switch will take place after three months in study phase 1 and a four-week break. At this point, it should be emphasized that the pharmacological treatment in both groups with antiseizure medication (ASM) will remain unaffected by study participation. It will be continued accordingly and, as usual, adjusted or increased depending on seizure frequency. This means that standard treatment will be ensured for both groups at all times.

To assess the independent variables (seizure frequency, serum ketosis), both groups will regularly measure blood ketone body concentrations using ketone test strips. Both groups will measure ketosis once per week, 30 minutes before the shake (IG) or the first meal (CG), and 30 minutes after the shake or the first meal. The recorded ketone values will be documented accordingly (see appendix). In addition, all participants will record their seizure frequency daily using the provided seizure diary (see appendix). Serum ketosis will also be assessed during study visits at our university outpatient clinic via blood sampling (see section blood sampling). Seizure frequency will also be assessed there.

Assessment of the dependent variables (microbiome changes, effects on neuronal networks, immune response, assessment of self-efficacy and quality of life) will be operationalized as follows:

Microbiome changes

To assess microbiome changes, participants will provide stool samples at four time points (end of baseline for visit T0.1, T1.2, end of washout period for visit T0.2, T2.2) using appropriate sterile containers that will be provided. These samples will be used for culture-independent, DNA-based sequencing of bacterial ribosomal 16S rRNA. In the present study, alpha and beta diversity of the gastrointestinal microbiome will be examined in particular.

Neuronal networks

To assess neuronal networks, a cerebral MRI (3T MRI including DTI and resting-state fMRI) and EEG will be performed at three time points (baseline, T2.1, T2.2).

Blood sampling

Different blood samples will be collected at different time points. In total, seven blood draws will be performed within the study. In routine care at the university outpatient clinic for epilepsy patients, so-called “small routine laboratory panels,” including antiseizure medication levels, are typically performed. Since participants are enrolled in a study, the additional blood draws (referred to as “large blood sampling” including immunological status) constitute an additional burden beyond routine clinical care. Participants will be informed about this.

Content of “small” blood sampling (baseline, T1.1, T2.1, T0.2):

• Complete blood count, electrolytes, TSH, CRP, kidney function parameters, liver function parameters (routine laboratory panel)

• Drug levels

• Lipid profile (HDL, LDL, total cholesterol, triglycerides)

• Ketone bodies (acetoacetate, β-hydroxybutyrate)

• MCTs (medium-chain triglycerides; C8 and C10)

Content of “large” blood sampling (T0.1, T1.2, T2.2) including immunological status:

• Differential blood count including immunological status (especially lymphocyte differentiation)

• Electrolytes, TSH, CRP, kidney function parameters, liver function parameters (routine laboratory panel)

• Drug levels

• Lipid profile (HDL, LDL, total cholesterol, triglycerides)

• Ketone bodies (acetoacetate, β-hydroxybutyrate)

• MCTs (medium-chain triglycerides; C8 and C10)

Questionnaires

At time points T0.1, T1.1, and T1.2 as well as T0.2, T1.2, and T2.2, short questionnaires will additionally be used to assess aspects of quality of life and self-efficacy. The corresponding questionnaires are provided in the appendix.

**19. Discontinuation Criteria**

For individual study participants: yes. Participants may withdraw from the study at any time and without providing reasons, without any disadvantages arising from doing so.

For termination of the entire study: no.

**E Data**

**20.** **Data collection.**

All participants will be pseudonymized using a patient identification number assigned by random draw. Only the study leadership (S. Knake, W. Hahn) will have access to the participants’ real names.

**21.** **Storage and transfer**

Data will be stored in anonymized form on a separate server. A key list containing real names and contact details for scheduling follow-up appointments will be created and managed by a study coordinator.

**References (original protocol):**

1.	Fisher RS, Boas W van E, Blume W, et al. Epileptic Seizures and Epilepsy: Definitions Proposed by the International League Against Epilepsy (ILAE) and the International Bureau for Epilepsy (IBE). *Epilepsia*. 2005;46(4):470-472. doi:10.1111/j.0013-9580.2005.66104.x

2.	Kossoff EH, Zupec‐Kania BA, Auvin S, et al. Optimal clinical management of children receiving dietary therapies for epilepsy: Updated recommendations of the International Ketogenic Diet Study Group. *Epilepsia Open*. 2018;3(2):175-192. doi:10.1002/epi4.12225

3.	Mandel A, Ballew M, Eric Pina-Garza J, Stalmasek V, Clemens LH. Medical Costs are Reduced when Children with Intractable Epilepsy are Successfully Treated with the Ketogenic Diet. *J Am Diet Assoc*. 2002;102(3):396-398. doi:10.1016/S0002-8223(02)90091-X

4.	Lum GR, Olson CA, Hsiao EY. Emerging roles for the intestinal microbiome in epilepsy. *Neurobiol Dis*. 2020;135:104576. doi:10.1016/j.nbd.2019.104576

5.	Chatzikonstantinou S, Gioula G, Kimiskidis VK, McKenna J, Mavroudis I, Kazis D. The gut microbiome in drug‐resistant epilepsy. *Epilepsia Open*. 2021;6(1):28-37. doi:10.1002/epi4.12461

6.	Payne NE, Cross JH, Sander JW, Sisodiya SM. The ketogenic and related diets in adolescents and adults-A review: Ketogenic and Related Diets in Adolescents and Adults. *Epilepsia*. 2011;52(11):1941-1948. doi:10.1111/j.1528-1167.2011.03287.x

7.	Harvie M, Wright C, Pegington M, et al. The effect of intermittent energy and carbohydrate restriction *v* . daily energy restriction on weight loss and metabolic disease risk markers in overweight women. *Br J Nutr*. 2013;110(8):1534-1547. doi:10.1017/S0007114513000792

8.	Varady KA. Intermittent versus daily calorie restriction: which diet regimen is more effective for weight loss? *Obes Rev*. 2011;12(7). doi:10.1111/j.1467-789X.2011.00873.x

9.	Michalsen A, Li C. Fasting Therapy for Treating and Preventing Disease - Current State of Evidence. *Complement Med Res*. 2013;20(6):444-453. doi:10.1159/000357765

10.	Hartman AL, Rubenstein JE, Kossoff EH. Intermittent fasting: A “new” historical strategy for controlling seizures? *Epilepsy Res*. 2013;104(3):275-279. doi:10.1016/j.eplepsyres.2012.10.011

11.	Olson CA, Vuong HE, Yano JM, Liang QY, Nusbaum DJ, Hsiao EY. The Gut Microbiota Mediates the Anti-Seizure Effects of the Ketogenic Diet. *Cell*. 2018;173(7):1728-1741.e13. doi:10.1016/j.cell.2018.04.027

12.	Olson CA, Iñiguez AJ, Yang GE, et al. Alterations in the gut microbiota contribute to cognitive impairment induced by the ketogenic diet and hypoxia. *Cell Host Microbe*. 2021;29(9):1378-1392.e6. doi:10.1016/j.chom.2021.07.004

13.	Peng A, Qiu X, Lai W, et al. Altered composition of the gut microbiome in patients with drug-resistant epilepsy. *Epilepsy Res*. 2018;147:102-107. doi:10.1016/j.eplepsyres.2018.09.013

14.	Gong X, Liu X, Chen C, et al. Alteration of Gut Microbiota in Patients With Epilepsy and the Potential Index as a Biomarker. *Front Microbiol*. 2020;11:517797. doi:10.3389/fmicb.2020.517797

15.	Tagliabue A, Ferraris C, Uggeri F, et al. Short-term impact of a classical ketogenic diet on gut microbiota in GLUT1 Deficiency Syndrome: A 3-month prospective observational study. *Clin Nutr ESPEN*. 2017;17:33-37. doi:10.1016/j.clnesp.2016.11.003

16.	Khan MN, Khan SI, Rana MI, Ayyaz A, Khan MY, Imran M. Intermittent fasting positively modulates human gut microbial diversity and ameliorates blood lipid profile. *Front Microbiol*. 2022;13:922727. doi:10.3389/fmicb.2022.922727

17.	Mujica-Parodi LR, Amgalan A, Sultan SF, et al. Diet modulates brain network stability, a biomarker for brain aging, in young adults. *Proc Natl Acad Sci*. 2020;117(11):6170-6177. doi:10.1073/pnas.1913042117
